# The *Arabidopsis* phytohormone crosstalk network involves a consecutive metabolic route and circular control units of transcription factors that regulate enzyme-encoding genes

**DOI:** 10.1186/s12918-016-0333-9

**Published:** 2016-09-02

**Authors:** Xun Yue, Xing Guo Li, Xin-Qi Gao, Xiang Yu Zhao, Yu Xiu Dong, Chao Zhou

**Affiliations:** 1 State Key Laboratory of Crop Biology, College of Life Sciences, Shandong Agricultural University, Tai’an, Shandong 271018 China; 2 State Key Laboratory of Crop Biology, College of Information Sciences and Engineering, Shandong Agricultural University, Tai’an, Shandong 271018 China

**Keywords:** Phytohormone crosstalk, Metabolic route, Circular control units, *Arabidopsis*, Network, Pathway

## Abstract

**Background:**

Phytohormone synergies and signaling interdependency are important topics in plant developmental biology. Physiological and genetic experimental evidence for phytohormone crosstalk has been accumulating and a genome-scale enzyme correlation model representing the *Arabidopsis* metabolic pathway has been published. However, an integrated molecular characterization of phytohormone crosstalk is still not available.

**Results:**

A novel modeling methodology and advanced computational approaches were used to construct an enzyme-based *Arabidopsis* phytohormone crosstalk network (EAPCN) at the biosynthesis level. The EAPCN provided the structural connectivity architecture of phytohormone biosynthesis pathways and revealed a surprising result; that enzymes localized at the highly connected nodes formed a consecutive metabolic route. Furthermore, our analysis revealed that the transcription factors (TFs) that regulate enzyme-encoding genes in the consecutive metabolic route formed structures, which we describe as circular control units operating at the transcriptional level. Furthermore, the downstream TFs in phytohormone signal transduction pathways were found to be involved in the circular control units that included the TFs regulating enzyme-encoding genes. In addition, multiple functional enzymes in the EAPCN were found to be involved in ion and pH homeostasis, environmental signal perception, cellular redox homeostasis, and circadian clocks. Last, publicly available transcriptional profiles and a protein expression map of the *Arabidopsis* root apical meristem were used as a case study to validate the proposed framework.

**Conclusions:**

Our results revealed multiple scales of coupled mechanisms in that hormonal crosstalk networks that play a central role in coordinating internal developmental processes with environmental signals, and give a broader view of *Arabidopsis* phytohormone crosstalk. We also uncovered potential key regulators that can be further analyzed in future studies.

**Electronic supplementary material:**

The online version of this article (doi:10.1186/s12918-016-0333-9) contains supplementary material, which is available to authorized users.

## Background

Phytohormone (plant hormone) crosstalk refers to phytohormone synergy and signaling interdependency. It is the main mechanism for regulating plant growth and development in combination with cell type, developmental stage and environmental conditions [1, 2]. Elucidating the components, architecture, and mechanisms of phytohormone crosstalk and how it helps coordinate plant growth are important in understanding plant developmental biology.

Many molecular and physiological studies have revealed information on major phytohormones at the molecular level, including abscisic acid, auxin, brassinosteroid, cytokinin, ethylene, gibberellin, jasmonic acid, and salicylic acid [2, 3]. It is now clear that phytohormone crosstalk comprises multiple levels of coupled mechanisms, including transcriptional regulation, signal transduction, biosynthesis, degradation, sequestration, transport, and complex metabolic conversion [4]. Much physiological and genetic experimental evidence for phytohormone crosstalk at the biosynthetic and transcriptional levels has been reported. For example, ethylene and auxin can reciprocally regulate each other [5–7]. JA-responsive ethylene response factor 109 (ERF109) binds directly to GCC-boxes in the promoters of ASA1 and YUC2, and mediates crosstalk between JA signaling and auxin biosynthesis to regulate lateral root formation in *Arabidopsis* [8]. Hormone profiling and the expression data for genes that encode key enzymes in abscisic acid and jasmonate biosynthesis showed that, in desiccated *Arabidopsis* roots, some hormonal regulation took place at the biosynthesis level [9]. Analyses of biosynthetic and signaling mutants, and studies of the roles of exogenous phytohormones have revealed extensive crosstalk and signal integration among growth-regulating hormones [2–4]. However, although physiological and genetic experimental evidences for phytohormone crosstalk has accumulated, until now, no integrated molecular characterization of phytohormone crosstalk in plant developmental biology has been published.

Because of the complexity of the relationships involved, a global perspective of the framework and mechanisms of phytohormone crosstalk cannot be gained based on the action of only one or a few molecules. Therefore, novel modeling methodologies and advanced computational approaches are required to determine the connections among phytohormones. Metabolic pathways and networks are emerging as powerful resources for identifying crucial biomarkers responsible for metabolic characteristics. The internal structure of a metabolic network can help elucidate the global activation status between mRNAs and proteins, and the metabolic mechanisms in a plant [10–12]. A major characteristic of metabolic pathways and networks is regulatory flexibility, where enzymes that regulate metabolic synthesis in one pathway can also catalyze metabolic reactions in other metabolic pathways. A comprehensive analysis of the enzymes involved in phytohormone metabolic pathways may help provide insights into the functional implications of phytohormone crosstalk.

In a previous study, we used the *Arabidopsis* metabolic pathway database (AraCyc 10.0) [13], which contains 540 pathways, 7127 enzymes, 3418 reactions, 3323 compounds, and 4225 citations, to construct a genome-scale enzyme correlation (GECN) model [14]. In this study, we used the GECN model to construct an *Arabidopsis* phytohormone crosstalk network model (named EAPCN) based on the phytohormone biosynthesis pathways in the *Arabidopsis* Hormone Database AHD2.0 [15]. The aim of this study was to use the EAPCN model to reveal the global mechanisms of phytohormone synergy and signaling interdependency at multiple levels in *Arabidopsis*.

## Methods

### Publicly available databases used in this study

The *Arabidopsis* metabolic pathway database AraCyc 10.0 (http://www.Arabidopsis.org/biocyc/) contains biochemical pathways that represent *Arabidopsis* metabolism (Additional file 1) [13]. AHD2.0 (http://ahd.cbi.pku.edu.cn/) is an updated version of the *Arabidopsis* Hormone Database containing information on eight major phytohormones in *Arabidopsis*: abscisic acid, auxin, brassinosteroids, cytokinin, ethylene, gibberellin, jasmonic acid, and salicylic acid [15]. The *Arabidopsis* interactome map contains approximately 6200 highly reliable interactions between approximately 2700 proteins [16]. The Plant Transcription Factor Database PlnTFDB (http://plntfdb.bio.uni-potsdam.de/v3.0/) contains approximately 2000 *Arabidopsis* genes that encode transcription factors (TFs).

The gene expression profile of the stele cells (three cells collected immediately after removing the stem cell niche) from *Arabidopsis* root tips was obtained from the Gene Expression Omnibus (http://www.ncbi.nlm.nih.gov/geo/) [GEO:GSE46226] [17]. The protein expression map of the *Arabidopsis* root (vasculature) came from Petricka et al. [18], and provided the identities and cell type-specific localization of nearly 2000 proteins from GeLC-MS/MS proteomic analysis.

### Genome-scale enzyme correlation network (GECN) model for *Arabidopsis*

The previously constructed GECN model [14] contains active information (enzymes, reactions, compounds, and citations) for individual metabolic pathways (Additional file 1). The nodes represent enzymes and edges represent two enzymes that interact with the same substrate.

### Construction of the *Arabidopsis* phytohormone crosstalk network (EAPCN) model

**Fig. 1:**
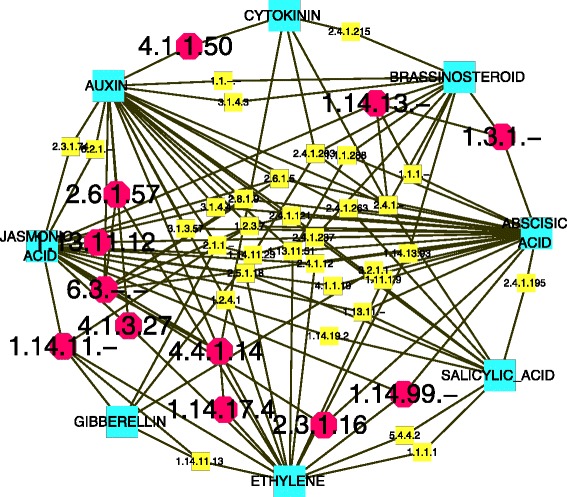
Structural connectivity of the enzyme-based *Arabidopsis* phytohormones crosstalk network (EAPCN) at the biosynthesis level. *Green nodes* represent eight phytohormones (abscisic acid, auxin, brassinosteroid, cytokinin, ethylene, gibberellin, jasmonic acid, and salicylic acid). The other nodes represent enzymes involved in synergistic or antagonistic crosstalk between the phytohormones. *Red nodes* represent enzymes for which there are experimental data (see Additional file 3, section I for details). *Yellow nodes* represent enzymes that are inferred to be involved in synergistic or antagonistic crosstalk. Edges represent nodes that exhibit either synergistic or antagonistic interactions
- Step 5: Network analysis and community analyses were used to investigate the characteristics of the systemic structure of the sub-interaction network (XML and XGMML format in Additional file 2).

The EAPCN model was constructed based on the GECN model as follows (Details are provided in Additional file 1):

- Step 1: Source files (TXT files) were downloaded from AraCyc 10.0 [13] and AHD2.0 [15].
- Step 2: The TXT file of information on eight major phytohormones in *Arabidopsis*, including pathways, enzymes, genes, reactions, compounds, and citations, was imported into an Oracle database platform.
- Step 3: The enzymes in AraCyc were mapped to the GECN model, and a sub-interaction network was constructed on an Oracle database platform using structured query language (SQL).
- Step 4: To infer antagonistic crosstalk between phytohormones, we included structural connectivity architecture at the biosynthesis level as another feature in the EAPCN model (Fig. 1). The EAPCN model provides a new platform for analyzing how collaborative mechanisms on multiple scales work from a global perspective.

### *Arabidopsis* TFs interactome network

To characterize the TFs that regulate the enzyme-encoding genes in the EAPCN model, we constructed a TF interactome network using the *Arabidopsis* TF data in PlnTFDB [19]. In the TFs interactome network, nodes represent TFs and edges indicate two TFs that have the same target gene. The interactome network was constructed on an Oracle database platform using SQL and visualized using the Cytoscape software [20] (XGMML, GML, SIF and NNF format in Additional file 2).

### Network topology analysis

NetworkAnalyzer is a Java plugin for Cytoscape, that compute specific parameters that describe the network topology [21]. We used NetworkAnalyzer to determine the number of connected pairs of nodes to examine the overall structure of the EAPCN model.

The centrality score of a node within a network crucially depends on the entire pattern of connections. A score indicates that the node may play key roles in controlling cellular functions. Centrality analysis was carried out on the EAPCN model using the cytoHubba plugin in Cytoscape [22]. We used 12 centrality parameters: Maximal Clique Centrality, Density of Maximum Neighborhood Component, Maximum Neighborhood Component, Degree, Edge Percolated Component, Bottleneck, Eccentricity, Closeness, Radiability, Betweenness, Stress, and Clustering Coefficient. All enzymes were sorted according to the 12 centrality parameters to identify highly connected nodes.

We used the graph clustering algorithm ClusterONE (clustering with overlapping neighborhood expansion), which is available as a plugin to Cytoscape [23], for the association analysis.

## Results and discussion

### Analysis of the EAPCN model revealed that enzymes localized at the highly connected nodes form consecutive metabolic pathways

**Fig. 2:**
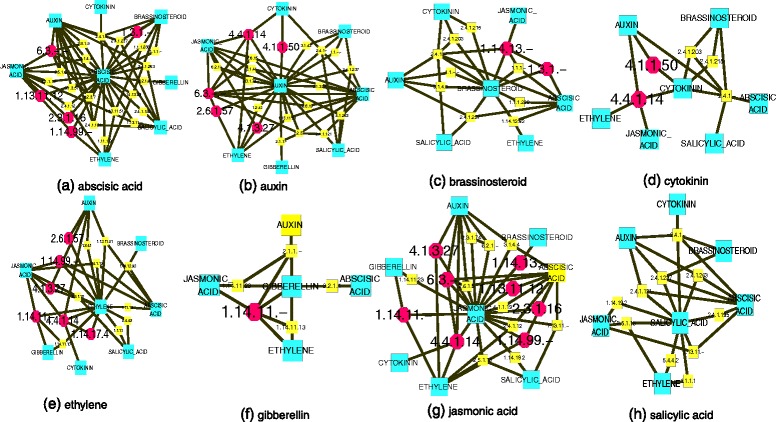
Detailed information of phytohormones and their crosstalk with other phytohormones in the EAPCN model. *Green nodes* represent eight phytohormones (abscisic acid, auxin, brassinosteroid, cytokinin, ethylene, gibberellin, jasmonic acid, and salicylic acid). The other nodes represent enzymes involved in synergistic or antagonistic crosstalk between the phytohormones. *Red nodes* represent enzymes for which there are experimental data (see Additional file 3, section I for details). *Yellow nodes* represent enzymes that are inferred to be involved in synergistic or antagonistic crosstalk. Edges represent nodes that exhibit either synergistic or antagonistic interactions

In the EAPCN model (Fig. 1), besides the nodes that represent the eight phytohormones (abscisic acid, auxin, brassinosteroids, cytokinin, ethylene, gibberellin, jasmonic acid and salicylic acid), other nodes represent enzymes that are involved in crosstalk between these phytohormones. For example, ACC SYNTHASE 10 (AT1G62960) encodes an aromatic amino acid transaminase (EC 2.6.1.57) that catalyzes the conversion of S-adenosylmethionine to 1-aminocyclopropane-1- carboxylic acid (ACC), which is the first committed and, in most instances, the rate-limiting step in ethylene biosynthesis [24]. Enzymes that regulate metabolic synthesis can be involved in other metabolic conversion pathways, and our analysis of the EAPCN showed that aromatic amino acid transaminase (EC 2.6.1.57) also catalyzed the conversion of keto-phenylpyruvate and L-glutamate to L-phenylalanine in the phenylalanine degradation III pathway [25]. The analysis also showed that Ligases (EC 6.3.-.-) could catalyze the conversion of L-phenylalanine to indole-3-acetyl- phenylalanine in the indole-3-acetic acid (IAA) degradation V pathway [26]. Because these enzymes (EC 2.6.1.57 and EC 6.3.-.-) form consecutive steps in a metabolic route to L-phenylalanine, it can be inferred that auxin and ethylene may be associated with inter-promotion or inter-restraint by EC 2.6.1.57 and EC 6.3.-.- at the biosynthesis level. As another example, mutation of BUSHY AND DWARF 2 (AT5G18930) has been shown to result in the loss-of-function of S-adenosylmethionine decarboxylase 4 (EC 4.1.1.50), which causes hyposensitivity to auxin and hypersensitivity to cytokinin [27]. This suggests that S-adenosylmethionine decarboxylase 4 may play a role in regulating synergistic or antagonistic crosstalk between auxin and cytokinin. Detailed information related to various phytohormones and their crosstalk with other phytohormones is shown in Fig. 2. Furthermore, some of the enzymes in the EAPCN model have been checked for consistency based on physiological and genetic experimental studies (see Additional file 3, section I for details).

It should be noted that the highly interconnected web of enzymes in the EAPCN model can be defined as a multimodal optimization problem in combination with cell type, developmental stage, and environmental conditions [28]. Nevertheless, the internal structure of the EAPCN model can be used to elucidate potential hubs of interaction and functional roles among phytohormones. To obtain detailed insights into the intrinsic properties of the network topology in the EAPCN model, we used the cytoHubba plugin of Cytoscape [22] for centrality analysis to determine the global properties of the EAPCN model. CytoHubba ranks connected enzymes according to their importance in a network. The data in Table 1 list the enzymes that were localized at the highly connected nodes in the EAPCN ranked according to 12 different centrality parameters.

**Table 1:** Centrality analysis to determine the global properties of the EAPCN model using CytoHubba plug-in in Cytoscape

MCC	DMNC	MNC	Degree	EPC	Bottleneck	Eccentricity	Closeness	Radiability	Betweenness	Stress	CC
6.3.-.-	1.2.3.7	6.3.-.-	6.3.-.-	6.3.-.-	2.4.1.-	2.4.1.237	6.3.-.-	2.4.1.-	2.4.1.-	2.4.1.-	1.14.17.4
1.2.3.7	2.8.1.9	1.2.3.7	2.4.1.-	2.4.1.-	1.13.11.51	2.4.1.203	2.4.1.-	2.4.1.237	4.4.1.14	2.4.1.237	2.8.1.9
2.4.1.-	2.4.1.203	2.4.1.-	4.4.1.14	4.4.1.14	1.14.13.-	6.3.-.-	2.4.1.237	6.3.-.-	2.4.1.237	4.4.1.14	1.14.11.13
1.14.11.-	1.14.11.13	1.14.11.-	2.4.1.237	2.4.1.237	1.14.11.-	1.14.11.23	4.4.1.14	4.4.1.14	6.3.-.-	6.3.-.-	2.3.1.74
1.14.13.-	2.1.1.-	1.14.13.-	1.2.3.7	1.2.3.7	1.1.-.-	1.14.13.-	1.2.3.7	1.13.11.51	1.14.11.-	1.13.11.51	6.2.1.-
4.4.1.14	2.4.1.121	2.6.1.5	2.1.1.-	2.6.1.5	1.13.11.-	4.4.1.14	1.13.11.51	1.2.3.7	1.14.11.23	2.4.1.121	1.2.3.7
2.1.1.-	4.4.1.14	2.8.1.9	1.14.11.-	1.13.11.51	4.4.1.14	1.13.11.12	2.6.1.5	2.4.1.121	2.1.1.-	1.14.13.93	2.6.1.5
2.6.1.5	2.3.1.74	2.4.1.203	1.14.11.23	2.4.1.121	1.13.11.12	2.5.1.18	2.4.1.121	2.6.1.5	1.13.11.51	2.4.1.263	6.3.-.-
2.4.1.237	6.2.1.-	1.14.11.13	1.14.13.-	1.14.11.23	2.5.1.18	1.2.4.1	3.1.4.4	2.4.1.263	1.14.13.-	4.1.3.27	2.4.1.203
2.4.1.203	1.13.11.51	2.1.1.-	2.4.1.203	2.1.1.-	1.2.4.1	1.14.99.-	1.14.99.-	3.1.4.4	1.14.13.93	2.6.1.5	2.4.1.121
2.4.1.121	1.14.11.23	2.4.1.121	1.13.11.51	3.1.4.4	1.14.99.-	1.2.3.7	2.4.1.12	1.14.99.-	2.4.1.121	3.1.4.4	1.1.1.288
1.14.11.23	1.1.1.288	4.4.1.14	2.4.1.121	1.14.13.-	2.4.1.237	2.8.1.9	1.14.13.93	2.4.1.12	1.2.3.7	1.14.99.-	2.6.1.57
2.8.1.9	2.6.1.57	2.3.1.74	2.6.1.5	2.8.1.9	1.2.3.7	2.4.1.12	2.4.1.263	1.14.13.93	3.2.1.1	2.4.1.12	1.1.1.-
1.14.11.13	1.1.1.-	6.2.1.-	2.5.1.18	1.14.11.-	2.8.1.9	1.14.11.13	1.14.13.-	4.1.3.27	2.4.1.263	1.14.11.-	1.3.1.-
1.13.11.51	1.3.1.-	1.13.11.51	1.14.99.-	4.1.3.27	2.4.1.203	1.14.13.93	4.1.3.27	1.14.13.-	2.4.1.203	1.13.11.-	2.1.1.-
2.3.1.74	1.14.17.4	1.14.11.23	2.8.1.9	2.4.1.12	2.4.1.12	1.13.11.51	1.14.11.-	1.14.11.-	4.1.3.27	2.5.1.18	1.14.11.-
6.2.1.-	6.3.-.-	1.1.1.288	2.4.1.12	1.14.99.-	1.14.11.13	2.3.1.16	1.14.11.23	1.14.11.23	1.14.99.-	1.14.13.-	1.14.13.-
2.5.1.18	2.4.1.-	2.6.1.57	1.14.11.13	2.6.1.57	1.14.13.93	2.4.1.-	2.1.1.-	2.6.1.57	2.4.1.12	1.2.3.7	4.4.1.14
1.14.99.-	1.14.11.-	1.1.1.-	1.14.13.93	1.14.13.93	2.3.1.16	2.4.1.195	1.13.11.-	2.8.1.9	2.5.1.18	2.1.1.-	1.13.11.51
2.4.1.12	1.14.13.-	1.3.1.-	2.4.1.263	1.13.11.-	2.4.1.195	1.11.1.9	2.8.1.9	1.13.11.-	1.13.11.-	1.14.11.23	2.4.1.-
1.14.13.93	2.6.1.5	1.14.17.4	1.1.1.288	2.4.1.263	1.11.1.9	2.1.1.-	2.6.1.57	2.1.1.-	2.6.1.5	2.6.1.57	1.14.11.23
2.4.1.263	1.13.11.12	1.13.11.12	2.6.1.57	2.4.1.203	2.1.1.-	1.14.11.-	1.1.1.288	1.1.1.288	3.1.4.4	1.11.1.9	1.13.11.12
1.1.1.288	2.5.1.18	2.5.1.18	4.1.3.27	2.3.1.74	2.4.1.121	2.4.1.121	2.5.1.18	2.5.1.18	1.1.1.288	2.8.1.9	2.5.1.18
2.6.1.57	1.2.4.1	1.2.4.1	1.13.11.-	2.5.1.18	6.3.-.-	1.1.-.-	2.3.1.74	3.1.3.57	1.1.1.-	3.1.3.57	1.2.4.1
4.1.3.27	1.14.99.-	1.14.99.-	1.1.1.-	1.1.1.-	1.14.11.23	2.4.1.215	6.2.1.-	1.11.1.9	1.3.1.-	1.2.4.1	1.14.99.-
1.13.11.-	2.4.1.237	2.4.1.237	2.3.1.74	6.2.1.-	2.4.1.215	4.1.1.19	3.1.3.57	1.13.11.12	2.6.1.57	1.1.1.-	2.4.1.237
1.1.1.-	2.4.1.12	2.4.1.12	3.1.4.4	1.3.1.-	4.1.1.19	2.4.1.263	1.11.1.9	2.3.1.16	1.14.11.13	1.3.1.-	2.4.1.12
3.1.4.4	1.14.13.93	1.14.13.93	6.2.1.-	1.1.1.288	2.4.1.263	3.2.1.1	1.1.1.-	4.1.1.19	1.11.1.9	1.1.1.288	1.14.13.93
1.3.1.-	2.3.1.16	2.3.1.16	1.3.1.-	3.1.3.57	3.2.1.1	1.1.1.288	1.3.1.-	2.3.1.74	4.1.1.50	2.4.1.195	2.3.1.16
1.13.11.12	2.4.1.195	2.4.1.195	1.13.11.12	4.1.1.19	1.1.1.288	2.6.1.57	1.13.11.12	6.2.1.-	1.2.4.1	2.3.1.74	2.4.1.195
1.2.4.1	1.11.1.9	1.11.1.9	1.2.4.1	1.11.1.9	2.6.1.57	4.1.3.27	2.3.1.16	1.1.1.-	1.1.-.-	6.2.1.-	1.11.1.9
2.3.1.16	1.1.-.-	1.1.-.-	2.3.1.16	1.13.11.12	4.1.3.27	1.1.1.1	4.1.1.19	1.3.1.-	3.1.4.3	1.1.-.-	1.1.-.-
2.4.1.195	2.4.1.215	2.4.1.215	2.4.1.195	1.14.11.13	1.1.1.1	2.6.1.5	1.2.4.1	2.4.1.203	2.4.1.195	3.1.4.3	2.4.1.215
1.11.1.9	4.1.1.19	4.1.1.19	1.11.1.9	2.3.1.16	2.6.1.5	1.13.11.-	2.4.1.203	1.2.4.1	1.1.1.1	1.14.19.2	4.1.1.19
1.1.-.-	2.4.1.263	2.4.1.263	1.1.-.-	3.1.4.3	1.1.1.-	1.1.1.-	2.4.1.195	2.4.1.195	5.4.4.2	2.4.1.203	2.4.1.263
2.4.1.215	3.2.1.1	3.2.1.1	2.4.1.215	1.14.19.2	5.4.4.2	5.4.4.2	3.2.1.1	3.2.1.1	2.8.1.9	1.13.11.12	3.2.1.1
4.1.1.19	4.1.3.27	4.1.3.27	4.1.1.19	1.1.-.-	2.3.1.74	2.3.1.74	1.1.-.-	1.1.-.-	3.1.3.57	2.3.1.16	4.1.3.27
3.2.1.1	1.1.1.1	1.1.1.1	3.2.1.1	1.2.4.1	4.1.1.50	4.1.1.50	3.1.4.3	3.1.4.3	1.14.19.2	4.1.1.19	1.1.1.1
1.1.1.1	1.13.11.-	1.13.11.-	1.1.1.1	1.14.17.4	3.1.4.3	3.1.4.3	1.14.19.2	1.14.19.2	2.3.1.74	3.2.1.1	1.13.11.-
5.4.4.2	5.4.4.2	5.4.4.2	5.4.4.2	2.4.1.195	3.1.4.4	3.1.4.4	1.14.11.13	1.14.17.4	6.2.1.-	1.1.1.1	5.4.4.2
4.1.1.50	4.1.1.50	4.1.1.50	4.1.1.50	5.4.4.2	3.1.3.57	3.1.3.57	1.14.17.4	1.14.11.13	2.4.1.215	5.4.4.2	4.1.1.50
3.1.4.3	3.1.4.3	3.1.4.3	3.1.4.3	3.2.1.1	6.2.1.-	6.2.1.-	4.1.1.50	1.1.1.1	1.13.11.12	4.1.1.50	3.1.4.3
3.1.3.57	3.1.4.4	3.1.4.4	3.1.3.57	1.1.1.1	1.14.19.2	1.14.19.2	1.1.1.1	5.4.4.2	2.3.1.16	1.14.11.13	3.1.4.4
1.14.19.2	3.1.3.57	3.1.3.57	1.14.19.2	4.1.1.50	1.3.1.-	1.3.1.-	5.4.4.2	4.1.1.50	4.1.1.19	2.4.1.215	3.1.3.57
1.14.17.4	1.14.19.2	1.14.19.2	1.14.17.4	2.4.1.215	1.14.17.4	1.14.17.4	2.4.1.215	2.4.1.215	1.14.17.4	1.14.17.4	1.14.19.2

We have used twelve centrality indices: Maximal Clique Centrality (MCC), Density of Maximum Neighborhood Component (DMNC), Maximum Neighborhood Component (MNC), Degree, Edge Percolated Component (EPC), Bottleneck, Eccentricity, Closeness, Radiability, Betweenness, Stress and Clustering Coefficient (CC) to check which one appears as highly connected nodes. All enzymes have been sorted by default parameters according to 12 different centrality parameters, EC numbers of the enzymes are listed

**Fig. 3:**
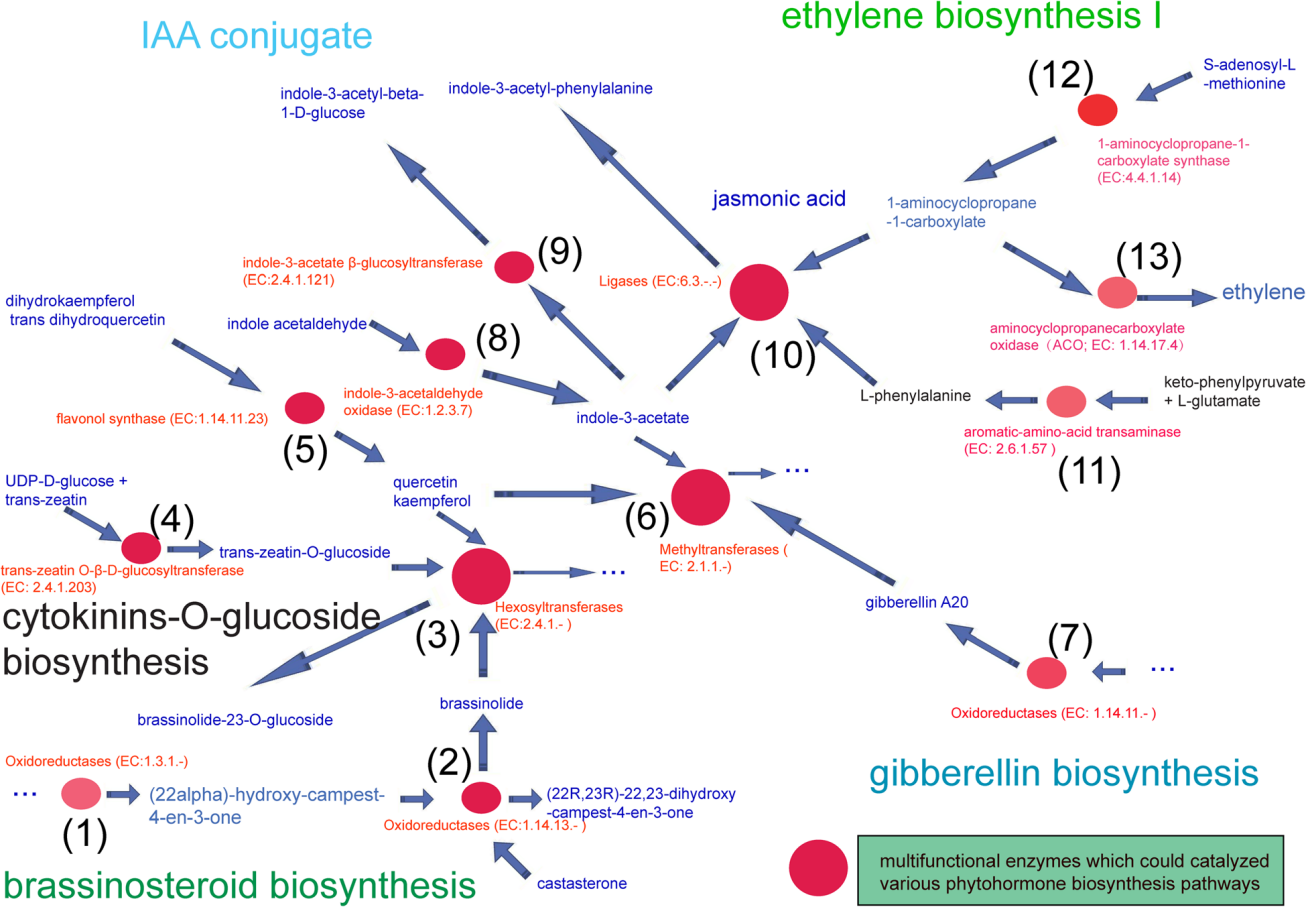
Consecutive metabolic route of phytohormone biosynthesis pathways in the EAPCN model. *Red circle nodes* represent the enzymes, *green nodes* represent the metabolites between the interactions among enzymes

Importantly, when ClusterONE was used for association analysis [23], we detected particular subsets of controlling sets in which enzymes localized at the highly connected nodes in the EAPCN made up a consecutive metabolic route (Fig. 3) with the following steps:

Oxidoreductases (EC 1.3.1.-) catalyze the conversion to (22α)-hydroxy-campest-4-en-3-one in the brassinosteroid biosynthesis II pathway.
(22α)-hydroxy-campest-4-en-3-one is catalyzed by oxidoreductases (EC 1.14.13.-) in the same pathway. Oxidoreductases (EC 1.14.13.-) were also found to catalyze the conversion of castasterone to brassinolide in the brassinosteroid biosynthesis I pathway.
Brassinolide is catalyzed by hexosyltransferases (EC 2.4.1.-) in the brassinosteroid inactivation pathway. Hexosyltransferases (EC 2.4.1.-) are multifunctional enzymes that may be involved in various phytohormone biosynthesis pathways. Our analysis of the EAPCN showed that hexosyltransferases (EC 2.4.1.-) can also catalyze the conversion of trans-zeatin-O-glucoside in the cytokinin 7-N-glucoside biosynthesis pathway, the conversion of kaempferol in the kaempferol glucoside biosynthesis pathway, and the conversion of quercetin in the quercetin glucoside biosynthesis pathway.
Trans-zeatin O-β-D-glucosyltransferase (EC 2.4.1.203) catalyzes the conversion of UDP-D-glucose and trans-zeatin to trans-zeatin-O-glucoside in the cytokinin-O-glucoside biosynthesis pathway.
Flavonol synthase (EC 1.14.11.23) catalyzes the conversion of dihydrokaempferol to kaempferol and the conversion of trans-dihydroquercetin to quercetin in the flavonol biosynthesis pathway.
Methyltransferases (EC 2.1.1.-) are also multifunctional enzymes. Our analysis showed that quercetin was catalyzed by methyltransferases (EC 2.1.1.-) in the quercetin sulfate biosynthesis pathway. Methyltransferases (EC 2.1.1.-) were also shown to catalyze the conversion of gibberellin A20 and S-adenosyl-L-methionine to gibberellin A20 methyl ester in the gibberellin inactivation II (methylation) pathway, the conversion of kaempferol in the kaempferol glucoside biosynthesis pathway, and the conversion of indole-3-acetate and S-adenosyl-L-methionine to methyl indole-3-acetate in the S-adenosyl-L-methionine cycle II pathway.
Oxidoreductases (EC 1.14.11.-) catalyze the conversion to gibberellin A20 in the gibberellin biosynthesis III (early C-13 hydroxylation) pathway.
Indole-3-acetaldehyde oxidase (EC 1.2.3.7) catalyzes the conversion of indole acetaldehyde to indole-3-acetate in the IAA biosynthesis I pathway.
Indole-3-acetate β-glucosyltransferase (EC 2.4.1.121) catalyzes the conversion of UDP-D-glucose and indole-3-acetate to indole-3-acetyl-beta-1-D-glucose in the superpathway of IAA conjugate biosynthesis.
Ligases (EC 6.3.-.-) catalyze the conversion of indole-3-acetate and L-phenylalanine to indole-3-acetyl-phenylalanine in the IAA degradation V pathway. Our analysis also found that ligases (EC 6.3.-.-) converted ACCin the jasmonoyl-amino acid conjugates biosynthesis I pathway.
Aromatic-amino-acid transaminase (EC 2.6.1.57) catalyzes the conversion of keto-phenylpyruvate and L-glutamate to L-phenylalanine in the phenylalanine degradation III pathway.
1-aminocyclopropane-1-carboxylate synthase (EC 4.4.1.14) catalyzes S-adenosyl-L-methionine to ACC in the ethylene biosynthesis I pathway.
Aminocyclopropanecarboxylate oxidase (ACO; EC 1.14.17.4) catalyzes L-ascorbate and ACC to ethylene in the ethylene biosynthesis I pathway.

The subsets of controlling sets containing enzymes localized at highly connected nodes in the EAPCN can form a consecutive metabolic route, indicating that intracellular hormone homeostasis and concentrations depend largely on the interaction of specific phytohormone combinations rather than on the independent activities of individual hormones. In particular, the consecutive metabolic route of various phytohormone biosynthesis pathways in the EAPCN model may be interlinked to mediate phytohormone synergy and signaling interdependency. These results will help to increase our understanding of phytohormone crosstalk at a global level and provide insights into how homeostasis and concentration levels of phytohormones influence plant growth and development.

### The homeostasis between auxin and cytokinin plays key roles in controlling cellular functions of phytohormone crosstalk

**Fig. 4:**
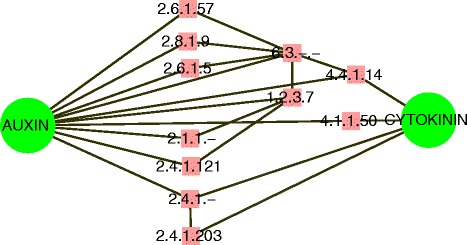
Eleven enzymes in the interaction between auxin and cytokinin of the AEEPBCN model

Literature evidence has shown that the phytohormone homeostasis between auxin and cytokinin has a strong impact on plant growth and development [2, 3]. In the AEEPBCN model, we detected 11 enzymes involved in the interaction between auxin and cytokinin, including, EC 2.4.1.-, EC 2.4.1.12, EC 2.4.1.121, EC 2.4.1.203, EC 2.1.1.-, EC 2.6.1.57, EC 2.6.1.5, EC 1.2.3.7, EC 4.4.1.14, EC 4.1.1.50, EC 2.8.1.9 and EC 6.3.-.- (Fig. 4). Importantly, most of the enzymes that regulate the interaction between auxin and cytokinin are in the consecutive metabolic route in the EAPCN model. In particular, hexosyltransferase (EC 2.4.1.-), which is multifunctional enzymes that are involved in the homeostasis of various phytohormone biosynthesis pathways, was located in step 3 of the consecutive metabolic route. A trans-zeatin-glucosyl-transferase (EC 2.4.1.203), which catalyzes the conjugation of glucose to cytokinin (UDP-glucose + trans-zeatin → UDP + O-glucosyl-trans-zeatin) and therefore regulates cytokinin activity, was located in step 4. Indole-3-acetaldehyde oxidase (EC 1.2.3.7), which catalyzes the last step of auxin biosynthesis (Indole-3-acetaldehyde → Indole-3-acetic acid), was located in step 8. An UDP-glycosyltransferase (EC 2.4.1.121), which conjugates glucose to IAA (UDP-glucose + (indol-3-yl)acetate → UDP + O-(indol-3-yl) acetyl-beta-D-glucose), was located in step 9. Additionally, methyltransferases (EC 2.1.1.-) were located in step 6, ligases (EC 6.3.-.-) were located in step 10, aromatic-amino -acid transaminase (EC 2.6.1.57) was located in step 11, and 1-aminocyclopropane-1 -carboxylate synthase (EC 4.4.1.14) was located in step 12. A detailed molecular and physiological study revealed that the loss-of-function of S-adenosylmethionine decarboxylase 4 (EC 4.1.1.50) causes hyposensitivity to auxin and hypersensitivity to cytokinin [27]. It is conceivable that the homeostasis between auxin and cytokinin plays key roles in controlling the cellular functions of phytohormone crosstalk.

### Subsets of TFs regulating enzyme-encoding genes in the EAPCN form circular control units that integrate consecutive metabolic pathways of phytohormone crosstalk

Transcriptional regulation is a common mechanism for integrating diverse phytohormone signals to regulate plant development. Phytohormones mostly influence the developmental process by modifying TF activity, which dynamically alters the transcriptome and leads to enzyme and metabolic changes. We constructed a TF interactome network using the *Arabidopsis* TF data in PlnTFDB [19] to characterize the TFs that regulate enzyme-encoding genes in the EAPCN model.

**Fig. 5:**
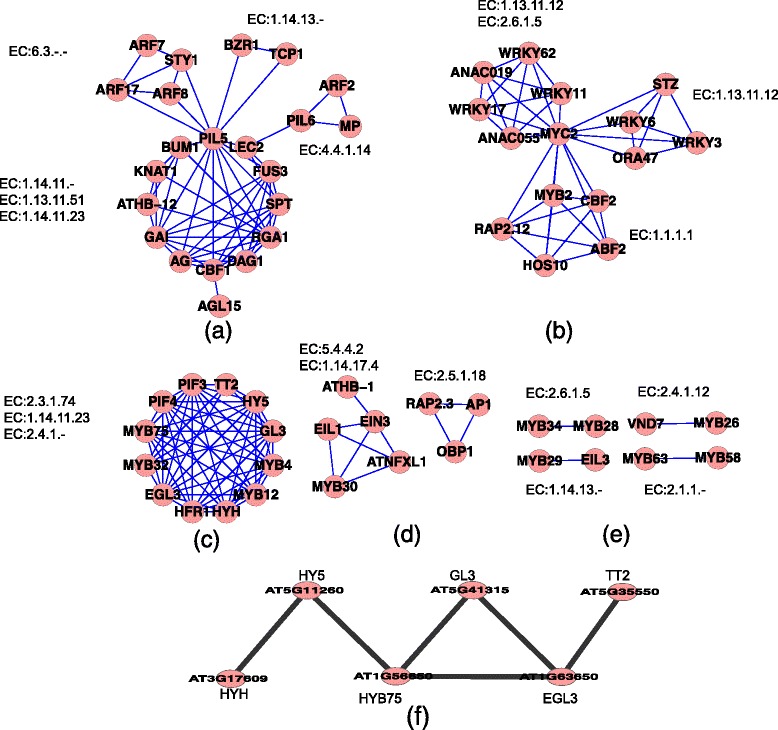
Circular control units of transcription factors (TFs) that regulate genes encoding enzymes in the EAPCN. Nodes represent the TFs, edges indicate two TFs that have the same target gene. **a**–**e** Circular control units of TFs that regulate genes that encode enzymes in EAPCN (see Additional file 3, section II for details), **f** Consecutive protein–protein interaction route in circular control unit C

As shown in Fig. 5, the network of TFs that regulate enzyme-encoding genes in the EAPCN has internal loop structures that may act as circular control units as follows (see Additional file 3, section II for details):

- A circular control unit, including PIL5 (AT2G20180), FUS3 (AT3G26790), RGA1 (AT2G01570), KNAT1 (AT4G08150), BUM1 (AT1G62360), AG (AT4G18960), DAG1 (AT3G61850), CBF1 (AT4G25490), SPT (AT4G36930), LEC2 (AT1G28300), AGL15 (AT4G36930), ATHB-12 (AT3G61890), and GAI (AT1G14920), regulates genes encoding the gibberellin oxidases (EC 1.14.11.-) GA3OX2 (AT1G80340), GA20OX1 (AT4G25420, EC 1.14.11.23), GA2OX4 (AT1G47990), and GA3OX1 (AT1G15550), and the epoxycarotenoid dioxygenase (EC 1.13.11.51) NCED9 (AT1G78390). A circular control unit, including PIL5 (AT2G20180), ARF8 (AT5G37020), STY1 (AT3G51060), ARF17 (AT1G77850), and ARF7 (AT5G20730), regulates genes encoding the IAA-amido synthetases (EC 6.3.-.-) GH3.5 (AT4G27260) and GH3.17 (AT1G28130), and NCED9 (EC 1.13.11.51) (AT1G78390). A circular control unit, including TCP1 (AT1G67260), PIL5 (AT2G20180), and BZR1 (AT1G75080), regulates genes encoding DWF4 (EC 1.14.13.-) (AT3G50660) and NCED9 (EC 1.13.11.51) (AT1G78390). A circular control unit, including MP (AT1G19850), PIL6 (AT3G59060), ARF2 (AT5G62000), and LEC2 (AT1G28300), regulates genes encoding the 1-aminocyclopropane-1-carboxylate synthase (EC 4.4.1.14) ACS8 (AT4G37770) and GA3OX2 (EC 1.14.11.-) (AT1G80340). In the EAPCN model, the enzymes that were found to be regulated by circular control units (i.e., EC 1.14.11.-, EC 1.13.11.51, EC 1.14.11.23, EC 6.3.-.-, EC 1.14.13.-, and EC 4.4.1.14) were all localized at hubs of interaction between phytohormones. In particular, PIL5 (AT2G20180) and LEC2 (AT1G28300) most likely act as hubs in the circular control unit structure. Previous physiological and genetic experimental studies have demonstrated that PIL5 inhibits seed germination not just through gibberellic acid and abscisic acid, but also by coordinating hormone signals and modulating cell wall properties in imbibed seeds [29]. ERF022–LEC2 interaction has been shown to be involved in the auxin–ethylene crosstalk that operates in somatic embryogenesis induction [30].
- A circular control unit, including WRKY62 (AT5G01900), WRKY17 (AT2G24570), ANAC019 (AT1G52890), MYC2 (AT1G32640), ANAC055 (AT3G15500), and WRKY11 (AT4G31550), regulates genes encoding (EC 1.13.11.12) LOX2 (AT3G45140) and TAT3 (EC 2.6.1.5) (AT2G24850). A circular control unit, including STZ (AT1G27730), ORA47 (AT1G74930), MYC2 (AT1G32640), WRKY6 (AT1G62300), and WRKY3 (AT2G03340), regulates a gene encoding LOX3 (EC 1.13.11.12) (AT1G17420). A circular control unit, including ABF2 (AT1G45249), HOS10 (AT1G35515), MYB2 (AT2G47190), CBF2 (AT4G25470), RAP2.12 (AT1G53910), and MYC2 (AT1G32640), regulates a gene encoding ADH1 (EC 1.1.1.1) (AT1G77120). MYC2 (AT1G32640) most likely acts as a hub in the circular control structure.
- A circular control unit, including TT2 (AT5G35550), TT4 (AT5G13930), MYB4 (AT4G38620), MYB12 (AT2G47460), MYB32 (AT4G34990), HFR1 AT1G02340), HY5(AT5G11260), PIF3 (AT5G13930), PIF4 (AT2G43010), HYH(AT3G17609), EGL3 (AT1G63650), GL3 (AT5G41315), and MYB75 (AT1G56650), regulates genes encoding the flavonol synthase (EC 1.14.11.23) LDOX (AT4G22880), naringenin-chalcone synthase (EC 2.3.1.74) TT4 (AT5G13930), and hexosyltransferase (EC 2.4.1.-) UGT78D2 (AT5G17050). In the EAPCN model, the enzymes regulated by circular control units (i.e., EC 1 .14.11.23, EC 2.3.1.74 and EC 2.4.1.-) were all localized at hubs of interaction between phytohormones.
- A circular control unit, including MYB30 (AT3G28910), EIN3 (AT3G20770), EIL1 (AT2G27050), ATNFXL1 (AT1G10170), and ATHB-1 (AT3G01470), regulates genes encoding EDS16 (EC 5.4.4.2) (AT1G74710) and ATACO1 (EC 1.14.17.4) (AT2G19590). A circular control unit, including OBP1 (AT3G50410), RAP2.3 (AT3G16770), and AP1 (AT1G69120), regulates a gene encoding GSTF8 (EC 2.5.1.18) (AT2G47730).
- MYB34 (AT5G60890) and MYB28 (AT5G61420) regulate a gene encoding SUR1 (EC 2.6.1.5) (AT2G20610). MYB26 (AT3G13890) and VND7 (AT1G71930) regulate a gene encoding IRX3 (EC 2.4.1.12) (AT5G17420). MYB29 (AT5G07690) and EIL3 (AT1G73730) regulate a gene encoding CYP79F2 (EC 1.14.13.-) (AT1G16400). MYB58 (AT1G16490) and MYB63 (AT1G79180) regulate a gene encoding ATOMT1 (EC 2.1.1.-) (AT5G54160).

Importantly, the enzymes regulated by these internal circular control units were all localized in the consecutive metabolic route in the EAPCN model. For example, oxidoreductases (EC 1.14.13.-) in step 2, hexosyltransferases (EC 2.4.1.-) in step 3, flavonol synthase (EC 1.14.11.23) in step 5, methyltransferases (EC 2.1.1.-) in step 6, oxidoreductases (EC 1.14.11.-) in step 7, ligases (EC 6.3.-.-) in step 10, 1-aminocyclopropane-1-carboxylate synthase (EC 4.4.1.14) in step 12, and ACO (EC 1.14.17.4) in step 13 (Fig. 3). These findings suggest that transcriptional co-repressors and adaptors assemble in a modular way to integrate simultaneous inputs from several phytohormone pathways, implying that they play central roles in this process. The circular control units of TFs that regulate enzyme-encoding genes in the EAPCN may act as the integrating mechanism to modulate consecutive metabolic pathways. It has been shown that the TFs, ARF2 [31], EIN3 [32], DWF4 [33], MYC2 [34], EGL3 and GL3 (bHLH factors), GL1 (MYB factor) [35], and HY5 [36] are activated by the cooperative action of phytohormone signaling pathways in the regulation of cellular activities including elongation, cell division and differentiation, organogenesis, pattern formation, reproduction, and responses to abiotic and biotic stresses. Based on the interaction information from a proteome-wide binary protein–protein interaction map of *Arabidopsis* [16], we investigated the specific protein interactions in the circular control units of EAPCN. We found that a particular consecutive protein–protein interaction route was present in circular control unit C (Fig. 5f), HYH (AT3G17609), HY5 (AT5G11260), MYB75 (AT1G56650), GL3 (AT5G41315), EGL3 (AT1G63650), and TT2 (AT5G35550), that regulates the genes encoding the flavonol synthase (EC 1.14.11.23) LDOX (AT4G22880), the naringenin-chalcone synthase (EC 2.3.1.74) TT4 (AT5G13930), and the hexosyltransferase (EC 2.4.1.-) UGT78D2 (AT5G17050). We also found that distinct TFs target multiple genes that encode enzymes involved in phytohormone biosynthesis, including EIN3 (AT3G20770), PIF3 (AT1G09530), PIL5 (AT2G20180), LEC2 (AT1G28300), MYC2 (AT1G32640), GL3 (AT5G41315), ARF8 (AT5G37020), ARF7 (AT5G20730), HY5 (AT5G11260), EGL3 (AT1G63650), and STY1 (AT3G51060) (see Additional file 3, section III for details).

### Downstream TFs in phytohormone signal transduction pathways are involved in the circular control units that regulate enzyme-encoding genes in the EAPCN

**Fig. 6:**
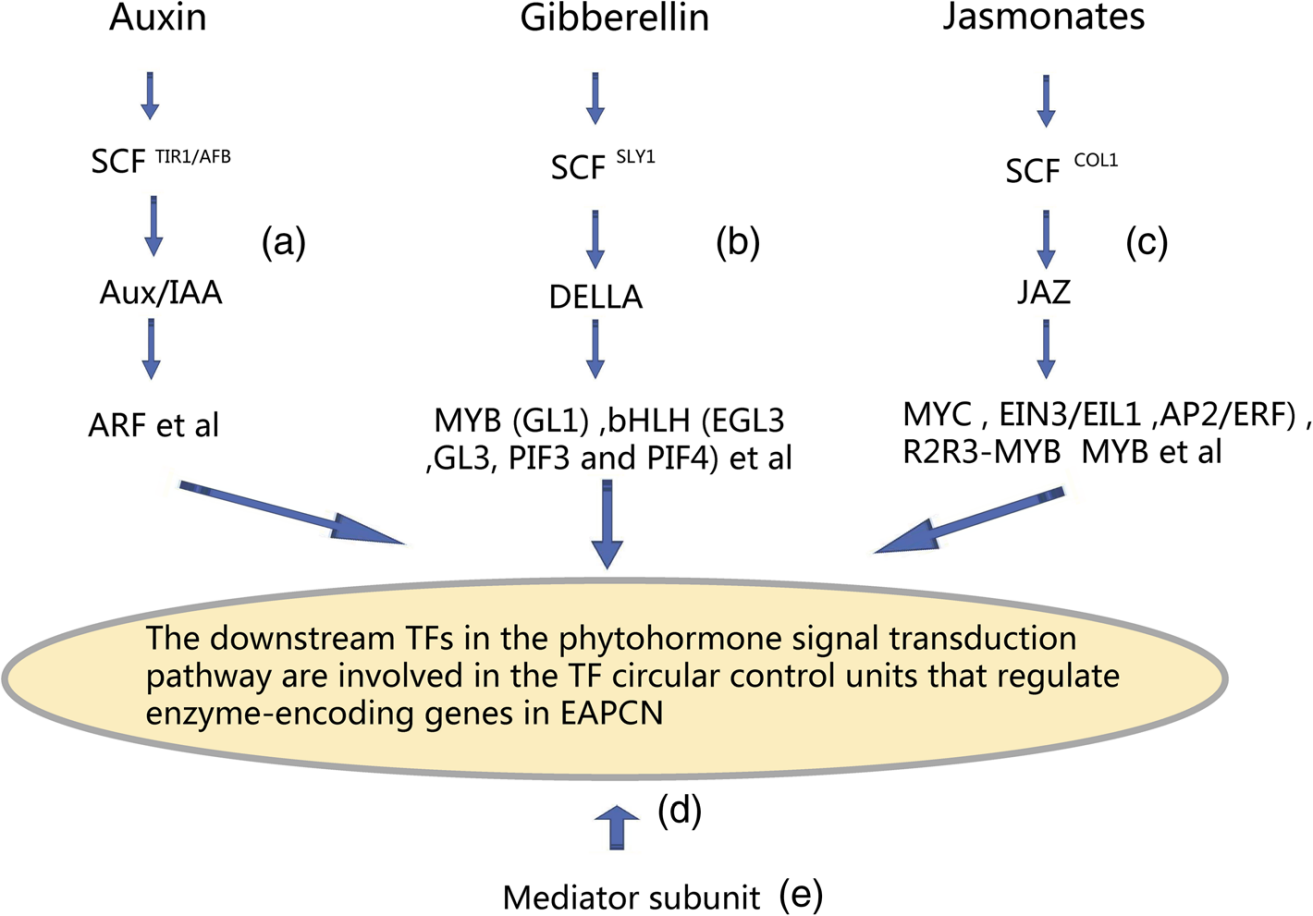
Simplified model for extracellular phytohormones that regulate enzyme-encoding genes or TFs in phytohormone biosynthesis by signal transduction pathways. **a** Auxin: SCF^TIR1/AFB^ complex. **b** Gibberellin: SCF^SLY1^ complex. **c** Jasmonates: SCF^COL1^ complex. **d** The downstream TFs in the phytohormone signal transduction pathway are involved in the TF circular control units that regulate enzyme-encoding genes in EAPCN. **e** Mediator complex regulates a wide range of signaling pathways by selectively interacting with specific TFs in phytohormone signal transduction pathways

Homeostasis and concentrations of phytohormones are both influenced by local phytohormone biosynthesis and transport from production sites to recipient tissues that require phytohormones for growth [3, 4]. Extracellular phytohormones may need before they can be converted to regulate genes encoding the enzymes required for endogenous phytohormone biosynthesis. Previous studies have elucidated signal transduction pathways from hormone biosynthesis to responses, and unique cellular components for the phytohormone-sensing and -response machinery of cells have been described [37]. For example, the SCF (Skp/Cullin/F-box) complex, an E3 ubiquitin ligase complex, plays a crucial role in regulating auxin, gibberellin, and jasmonic acid responses because it targets transcriptional repressor proteins for degradation upon perception of biologically active phytohormones [38–40]. In Fig. 6, a simplified model was used to describe extracellular phytohormones that are converted to regulate enzyme-encoding genes or TFs in phytohormone biosynthesis by signal transduction pathways. The auxin: SCF^TIR1/AFB^ complex, which consists of four subunits (TIR1/AFB, ASK1, CUL1, and RBX), regulates the Aux/IAA transcriptional repressors, and the ARF TFs [40] (Fig. 6a). The gibberellin: SCF^SLY1^ complex regulates DELLA, MYB (GL1), and bHLH (EGL3, GL3, PIF3, and PIF4) TFs, which are members of the WD-repeat/bHLH/ MYB complex [35, 38] (Fig. 6b). The jasmonates: SCF^COL1^ ubiquitin-ligase complex, which associates with AtCUL1, AtRbx1, and the Skp1-like proteins ASK1 and ASK2, regulates the JA ZIM-domain (JAZ) repressors, the bHLH subgroup IIIe factors MYC and EIN3/EIL1, bHLH subgroup IIId factors, APETALA2/ETHYLENE RESPONSE FACTOR (AP2/ERF), R2R3-MYB TFs, MYB, and the WD-repeat/bHLH/MYB transcription complex [35] (Fig. 6c).

In the EAPCN model, the auxin response factors ARF7 (AT5G20730) and ARF17 (AT1G77850) regulate a gene encoding IAA-amido synthetase GH3.5 (AT4G27260), and ARF8 (AT5G37020) regulates a gene encoding the GH3 enzyme (EC 6.3.-.-) GH3.17 (AT1G28130), which act in the crosstalk of abscisic acid, auxin, and jasmonic acid. ARF2 (AT5G62000) regulates a gene encoding the 1-aminocyclopropane-1-carboxylate synthase (EC 4.4.1.14) ACS8 (AT4G37770), which acts in the crosstalk of auxin, cytokinin, ethylene, and jasmonic acid. The downstream TFs in the gibberellin signal transduction pathway (i.e., EGL3, GL3, PIF3, and PIF4) regulate genes encoding the flavonol synthase (EC 1.14.11.23) LDOX (AT4G22880), the naringenin-chalcone synthase (EC 2.3.1.74) TT4 (AT5G13930), and the hexosyltransferase (EC 2.4.1.-) UGT78D2 (AT5G17050), which act in the crosstalk of abscisic acid, auxin, gibberellin, brassinosteroids, salicylic acid, cytokinin, and jasmonic acid. Similarly, the downstream TFs in the jasmonate signal transduction pathway, including MYC2, EIN3, EIL1, MYB2, MYB4, MYB12, MYB26, MYB28, MYB29, MYB30, MYB32, MYB34, MYB58, MYB63, and MYB75, regulate genes encoding various phytohormone biosynthetic pathways in the EAPCN model. Importantly, the downstream TFs in the phytohormone signal transduction pathway are all involved in the circular control units that regulate enzyme-encoding genes in the EAPCN (Fig. 6d). Thus, it can be inferred that extracellular phytohormones are controlled by signal transduction pathways to activate genes that encode enzymes involved in phytohormone biosynthesis, thereby regulating endogenous interconnected phytohormone homeostasis. These results will help in understanding the phytohormone-sensing and -response machinery, which serve as regulatory hubs to mediate crosstalk among the phytohormone signaling pathways.

The mediator complex is a multi-protein transcriptional co-activator complex that serves as a bridge between gene-specific TFs and the RNA polymerase machinery to regulate transcription. Chen et al. [41] reported that MED25 physically associates with MYC2 and exerts a positive effect on MYC2-regulated gene transcription in *Arabidopsis*. Recently, Wang et al. [42] showed that the mediator complex subunit MED16 regulated resistance to *Sclerotinia sclerotiorum* by governing both jasmonic acid/ethylene-mediated and WRKY33-activated defense signaling in *Arabidopsis*. These results suggest that the mediator complex regulates a wide range of signaling pathways by selectively interacting with specific TFs associated with phytohormone signal transduction pathways (Fig. 6e), and may play central roles from hormone perception to responses.

### Multiple functional redundancies in enzyme-based regulatory processes revealed in the EAPCN model

A major characteristic of metabolic pathways and networks is regulatory flexibility, which means that enzymes that regulate metabolic synthesis can also catalyze other metabolic conversion pathways and metabolism can progress through multiple metabolic pathways. We analyzed functional redundancy in enzyme-based regulatory processes to identify co-regulated biological processes in the EAPCN model and obtain a better understanding of how homeostasis and variable concentrations of phytohormones influence plant growth and development.

The first significant result was phospholipase D (EC 3.1.4.4), phospholipase C (EC 3.1.4.3), and inositol-1. 4-bisphosphate 1-phosphatase (EC 3.1.3.57) in the EAPCN model. It has been reported that phospholipase C produces two important secondary messenger molecules, inositol 1, 4, 5-trisphosphate and diacylglycerol, and that phospholipase D hydrolyzes phospholipids at the terminal phosphodiester bond and generates phosphatidic acid [43]. It has been shown that inositol 1, 4, 5-trisphosphate and phosphatidic acid together play vital roles in regulating a feedback loop from the cytosol to the plasma membrane toregulate Ca^2+^ levels [44, 45]. Recently, it was reported that calcium mediates the formation of stable CIPK–CBL complexes, which regulate the phosphorylation state and activity of various ion transporters involved in the maintenance of cell ion homeostasis in plants [46, 47]. The maintenance of organelle-specific ion and pH homeostasis has been shown to be a cell-intrinsic phenomenon [48]. It can be speculated that the cell microenvironment is governed by ion and pH homeostasis, which is regulated by intracellular phytohormone crosstalk and therefore closely linked to rapid changes in gene expression, metabolic regulation, signaling, and cell behaviors.

Cellular redox homeostasis plays an important role in every aspect of plant biology. Accumulating evidence suggests that the redox signaling hub interfaces with the phytohormone network in response to environmental stress [49, 50]. Our second significant result was that, in the EAPCN model, major distinct enzymes are involved in glutathione redox reactions. For example, glutathione peroxidase (EC 1.11.1.9) regulates the crosstalk of ABA and ethylene, and glutathione transferase (EC 2.5.1.18) regulates the crosstalk of ethylene, jasmonic acid, and salicylic acid. Glutathione peroxidases (EC 1.11.1.9) are a major family of reactive oxygen species scavenging enzymes [51, 52]. Glutathione S-transferases (GSTs; EC 2.5.1.18) catalyze the nucleophilic conjugation of reduced tripeptide glutathione (GSH; g-Glu-Cys-Gly). This result suggests that redox changes in the glutathione pool can affect growth through phytohormone crosstalk.

Circadian clocks coordinate numerous biological events with the environment [53]. Recent studies in *Arabidopsis* have identified many clock components that regulate transcription in hormone signaling pathways. For example, the clock-controlled expression of the GA biosynthetic enzyme GA20ox1 and GA-INSENSITIVE DWARF1 (GID1, which encodes a GA receptor) contribute to a higher abundance of a GA–GID1 complex around dawn, which promotes degradation of DELLA TFs. DELLA TFs are crucial repressors of the GA signaling pathway and block PHYTOCHROME INTERACTING FACTOR4 (PIF4) activity by binding to the PIF4 DNA-binding domain [54]. In addition, PIF4 has been shown to influence auxin production, and PIF4 and PIF5 both affect auxin signaling downstream of biosynthesis [55]. Our third significant result was that, in the EAPCN, PIF3 and PIF4 are part of the circular control unit that regulates the genes encoding the flavonol synthase (EC 1.14.11.23) LDOX (AT4G22880), the naringenin-chalcone synthase (EC 2.3.1.74) TT4 (AT5G13930), and the hexosyltransferase (EC 2.4.1.-) UGT78D2 (AT5G17050), and PIF3 (AT1G09530) mediates multiple target genes of various phytohormone biosynthesis enzymes (see Additional file 3, section III for details). Thus, it seems likely that phytohormone crosstalk is coordinated with the circadian system.

Compartmentation of phytohormone biosynthesis pathways is the basis of metabolic complexity [56]. The fourth significant result of our study was that many of the enzymes (e.g., EC 1.3.1.-, EC 1.1.1.-, and EC 6.2.1.-) in the EAPCN model are localized in chloroplasts or mitochondria. Retrograde signaling of chloroplasts and mitochondria affects the transcriptional and translational machinery to influence nuclear gene expression [57]. Nuclear genes encoding chloroplast and mitochondria proteins also regulate the metabolic adjustment in response to changing environmental conditions [58]. Therefore, it seems that the spatial scale of the structural connectivity of the EAPCN model could be important for enzyme activities, phytohormone storage and transport, and different phytohormone metabolites related to the growth environment. Furthermore, phytohormone crosstalk may play a central role in coordinating internal developmental processes with environmental signals.

### An integrated model showing multiple levels of components, structural connectivity architecture, and the mechanism of phytohormone crosstalk from a global perspective

**Fig. 7:**
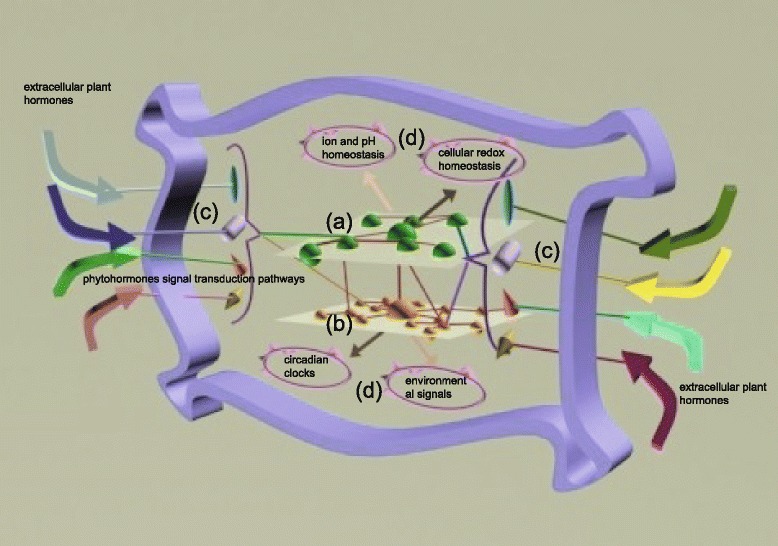
Integrated model showing multiple levels of collaborative mechanisms in the EAPCN from a global perspective. **a** Endogenous enzyme-based phytohormones biosynthesis crosstalk network. **b** Transcriptional regulatory module (i.e., TFs) that regulates enzyme-encoding genes related to phytohormones crosstalk. **c** Extracellular phytohormones regulate genes that encode enzymes involved in phytohormone biosynthesis through phytohormone signal transduction pathways. **d** Co-regulated biological processes in the phytohormone crosstalk network

Based on our results, we built an integrated model to demonstrate the multiple levels of collaborative mechanisms of phytohormone crosstalk from a global perspective (Fig. 7). The inferred model defines the multiple levels of components, structural connectivity architecture, and biological mechanisms. At the biosynthesis level, phytohormone crosstalk is controlled by sophisticated synergistic or antagonistic relationships between enzyme-based phytohormone biosynthesis pathways (Fig. 7a). The EAPCN, which was constructed to investigate the structural connectivity architecture of the various phytohormone biosynthesis pathways, revealed that the enzymes localized at highly connected nodes in the EAPCN formed a consecutive metabolic route. At the transcriptional level, hormone homeostasis isregulated by transcriptional and post-translational regulation of genes that encode enzymes involved in the phytohormone biosynthetic pathways (Fig. 7b). We found that the TFs that regulate genes encoding enzymes of the consecutive metabolic route formed circular control units, and extracellular phytohormones were converted by signal transduction pathways to regulate enzyme-encoding genes or TFs in various phytohormone biosynthesis pathways. Downstream TFs associated with the phytohormone signal transduction pathways were involved in the circular control units that regulate enzyme-encoding genes in the EAPCN (Fig. 7c). In addition, multiple functional redundancy in enzyme-based processes that regulate conversion complexities in the EAPCN model were involved in ion and pH homeostasis, environmental signals, cellular redox homeostasis, and circadian clocks (Fig. 7d).

### Transcriptional profiles and a protein expression map of the *Arabidopsis* root apical meristem support the proposed framework of phytohormone crosstalk

Phytohormone crosstalk is tightly constituted by the multiple scales of coupled mechanisms (i.e. transcriptional regulation, protein abundance over post-translational modifications, signal transduction, synthesis, degradation and metabolic conversion complexities et al.). It is now widely recognized that gene expression at the transcriptional level need not equate to protein expression at the protein level and certainly not at the enzyme activity level. Thus, to validate the proposed framework of phytohormone crosstalk, transcriptional profiles and a protein expression map of *Arabidopsis* root stele cells (vasculature) were used to analyze the expressed enzyme-encoding genes, TFs, and TFs target genes in the EAPCN model. As shown in Table 2, except for trans-zeatin O-β-D- glucosyltransferase (EC 2.4.1.203), enzyme-encoding genes in the consecutive metabolic route of the controlling sets in the EAPCN model were involved in *Arabidopsis* root vasculature. In particular, oxidoreductases (EC 1.3.1.-, EC 1.14.13.-), hexosyltransferases (EC 2.4.1.-), methyltransferases (EC 2.1.1.-), glutathione transferase (EC 2.5.1.18), alcohol dehydrogenase (EC 1.1.1.1), abscisic acid 8′-hydroxylase (EC 1.14.13.93), TT4 (EC 2.3.1.74), acyl-[acyl-carrier-protein] desaturase (EC 1.14.19.2) and cellulose synthase (EC 2.4.1.12) showed correlations between proteome and transcriptional profiles. In addition, we found that the gene expression profiles in the *Arabidopsis* root apical meristem (vasculature) had specific characteristics in the EAPCN model. For example, the genes encoding DWF4 (AT3G50660), EIL3 (AT1G73730), and BZR1 (AT1G75080) regulate genes encoding oxidoreductases (EC 1.14.13.-) in step 2 of the consecutive metabolic route. Genes encoding FT1 (AT2G03220), VRN1 (AT3G18990), and UGT78D2 (AT5G17050) regulate genes encoding hexosyltransferases (EC 2.4.1.-) in step 3. Genes encoding MYB32 (AT4G34990), HY5 (AT5G11260), FLS1 (AT5G08640), MYB12 (AT2G47460), RGA1 (AT2G01570), and GAI (AT1G14920) regulate genes encoding flavonol synthase (EC 1.14.11.23) in step 5. Genes encoding SPT (AT4G36930), MYB32 (AT4G34990), GA2OX6 (AT1G02400), CBF1 (AT4G25490), GAI (AT1G14920), DAG1 (AT3G61850), RGA1 (AT2G01570), DDF1 (AT1G12610), and HY5 (AT5G11260) regulate genes encoding oxidoreductases (EC 1.14.11.-) in step 7. Genes encoding AAO1 (AT5G20960) regulate genes encoding Indole-3-acetaldehyde oxidase (EC 1.2.3.7) in step 8. Genes encoding the auxin response factor ARF7 (AT5G20730), DFL1 (AT5G54510), and WES1 (AT4G27260) regulate genes encoding GH3 (EC 6.3.-.-) in step 10. Genes encoding the auxin response factor ACS6 (AT4G11280), MP (AT1G19850), ARF2 (AT5G62000), ACS2 (AT1G01480), and ACS8 (AT4G37770) regulate genes encoding 1-aminocyclopropane-1-carboxylate synthase (EC 4.4.1.14) in step 12. Finally, genes encoding EIN3 (AT3G20770), ATHB-1 (AT3G01470), and ATACO2 (AT1G62380) regulate genes encoding ACO (EC 1.14.17.4) in step 13 of the consecutive metabolic route. These results support the proposed framework of phytohormone crosstalk, and indicate that the consecutive metabolic route of controlling sets in the EAPCN model is involved in *Arabidopsis* root vasculature (except trans-zeatin O-β-D-glucosyltransferase (EC 2.4.1.203)). Further support for the core control structural and functional processes in phytohormone crosstalk could be provided using reverse genetic approaches to elucidate the biological functions of phytohormones, especially their roles in regulating cellular activities such as elongation, cell division and differentiation, organogenesis, pattern formation, reproduction, and responses to abiotic and biotic stress conditions.

**Table 2:** Transcriptional profiles and a protein expression map of enzyme-encoding genes, TFs, and TF target genes and enzymes in *Arabidopsis* root apical meristem (vasculature)

Enzyme	Expressed TFs, TF target genes	Expressed genes encoding enzymes	Protein expression	Crosstalk
(EC 1.3.1.-) (Step 1)^a^		AT3G55360	AT3G55360	Abscisic acid Brassinosteroid
EC 1.14.13.- (Step 2)^a^	DWF4(AT3G50660) EIL3(AT1G73730) BZR1(AT1G75080)	AT2G27690 AT5G04660 AT4G36380 AT5G38970	AT5G48000	Brassinosteroid Jasmonic acid
EC 2.4.1.- (Step 3)^a^	FT1(AT2G03220) VRN1(AT3G18990) UGT78D2(AT5G17050)	AT3G53160 AT3G21750 AT4G34138 AT1G73880 AT4G34135 AT1G78270 AT1G06000 AT2G30140 AT5G40390 AT2G28080	AT1G06000	Abscisic acid Auxin Brassinosteroid Cytokinin Salicylic acid
EC 1.14.11.23 (Step 5)^a^	MYB32(AT4G34990) HY5(AT5G11260) FLS1(AT5G08640) MYB12(AT2G47460) RGA1(AT2G01570) GAI(AT1G14920)	AT3G50210 AT3G49630 AT3G19010 AT4G16770		Gibberellin Jasmonic acid
EC 2.1.1.- (Step 6)^a^		AT3G63410 AT5G54160	AT3G63410	Auxin Gibberellin
EC 1.14.11.- (Step 7)^a^	SPT(AT4G36930) MYB32(AT4G34990) GA2OX6(AT1G02400) CBF1(AT4G25490) GAI(AT1G14920) DAG1(AT3G61850) RGA1(AT2G01570) DDF1(AT1G12610) HY5(AT5G11260)	AT1G14130		Ethylene Gibberellin Jasmonic acid
EC 1.2.3.7 (Step 8)^a^	AAO1(AT5G20960)	AT2G27150		Abscisic acid Auxin
EC 2.4.1.121 (Step 9) ^a^		AT4G15550		Abscisic acid Auxin Salicylic acid
EC 6.3.-.-: (Step 10)^a^	ARF7(AT5G20730) DFL1(AT5G54510) WES1(AT4G27260)	AT2G46370		Abscisic acid Auxin Jasmonic acid
EC 2.6.1.57 (Step 11)^a^		AT1G70560 AT1G62960 AT5G51690		Abscisic acid Auxin Jasmonic acid
EC 4.4.1.14 (Step 12)^a^	ACS6(AT4G11280) MP(AT1G19850) ARF2(AT5G62000) ACS2(AT1G01480) ACS8(AT4G37770)	AT5G51690 AT1G62960		Auxin Cytokinin Ethylene Jasmonic acid
EC 1.14.17.4 (Step 13)^a^	EIN3(AT3G20770) ATHB-1(AT3G01470) ATACO2(AT1G62380)	AT1G05010 AT1G77330		Ethylene
EC 2.5.1.18	OBP1(AT3G50410) GSTF8(AT2G47730) ANAC019(AT1G52890) AP2.3(AT3G16770)	AT2G30870 AT2G29440 AT2G29490 AT5G41210 AT1G75270 AT1G17170 AT1G27140 AT2G29480 AT2G30860 AT1G17190 AT4G19880 AT5G45020 AT5G41240 AT5G41220 AT5G44990 AT1G78340 AT2G29420 AT1G27130 AT2G29460 AT1G17180 AT2G29450 AT1G78380 AT2G29470 AT2G02390	AT2G30870 AT2G30860 AT2G47730 AT1G78380	Ethylene Jasmonic acid Salicylic acid
EC 1.1.1.1	MYC2(AT1G32640) RAP2.12 (AT1G53910) ADH1(AT1G77120) ABF2(AT1G45249) CBF2(AT4G25470) MYB2(AT2G47190	AT5G43940 AT5G63620 AT5G19440 AT5G24760	AT5G43940 AT5G19440	Ethylene Salicylic acid
EC 1.13.11.12	LOX3 (AT1G17420) MYC2 (AT1G32640) ORA47 (AT1G74930) STZ(AT1G27730) WRKY6(AT1G62300) WRKY3(AT2G03340) ANAC055(AT3G15500) ANAC019(AT1G52890) WRKY11(AT4G31550)	AT3G22400 AT1G67560		Abscisic acid Jasmonic acid
EC 1.14.11.13	GA2OX6(AT1G02400) DDF1(AT1G12610) CBF1(AT4G25490)	AT1G14130 AT1G14120		Ethylene gibberellin
EC 1.14.13.93	BZR1(AT1G75080) CPD(AT5G05690) DWF4(AT3G50660)	AT4G19230 AT5G45340 AT5G38970 AT4G36380	AT5G48000 AT5G05690	Abscisic acid Brassinosteroid Ethylene
EC 2.3.1.74	TT4(AT5G13930) MYB12(AT2G47460) HYH(AT3G17609)		AT5G13930	Auxin Jasmonic acid
EC 1.14.19.2		AT5G16240 AT2G43710 AT3G02630	AT3G02630 AT2G43710	Jasmonic acid Salicylic acid
EC 1.1.1.288		AT1G52340		Abscisic acid Brassinosteroid
EC 2.4.1.12	VND7(AT1G71930)	AT5G16910 AT3G03050 AT5G64740 AT5G09870 AT4G32410 AT4G39350 AT5G05170 AT2G21770 AT1G55850 AT2G33100 AT1G71930	AT4G32410 AT5G64740 AT5G05170	Abscisic acid Ethylene Jasmonic acid
EC 2.4.1.215		AT3G53160 AT1G78270 AT2G28080 AT2G30140		Brassinosteroid Cytokinin
EC 2.4.1.237		AT1G73880 AT4G34138 AT2G15490 AT4G34135 AT5G59580 AT5G59590 AT1G07250		Abscisic acid Auxin Brassinosteroid Salicylic acid
EC 2.4.1.263		AT4G34138 AT4G15550 AT4G34135 AT1G07260 AT2G30150 AT2G31750 AT1G07250		Abscisic acid Auxin Salicylic acid
EC: 2.4.1.195		AT1G05680 AT2G30150 AT2G31750 AT1G24100		Abscisic acid Salicylic acid

^a^Indicates consecutive metabolic route of controlling sets in the EAPCN model (see Fig. 3).

## Conclusions

Plant growth and development is influenced both by local phytohormone biosynthesis and the transport from production sites to recipient tissues that require phytohormones for growth. The final effect of an individual hormone is established by hormonal pathways that are interconnected through a complex network of interactions and feedback regulations. Representing the complexity of the relationships involved in phytohormone crosstalk is difficult. The EAPCN model presented here revealed that several phytohormone pathways are interlinked at the hormone biosynthesis level. Our results revealed multiple scales of coupled mechanisms in hormonal crosstalk networks that play a central role in coordinating internal developmental processes with environmental signals. Our main observations were: (i) enzymes localized at highly connected nodes of the EAPCN form a consecutive metabolic route; (ii) TFs regulating genes that encode enzymes in the consecutive metabolic route form circular control units that act at the transcriptional level; (iii) downstream TFs of phytohormone signal transduction pathways are also involved in the circular control units of TFs that regulate enzyme-encoding genes; and (iv) multi-functional enzymes in the EAPCN are involved in maintaining ion and pH homeostasis, coordinating internal developmental processes with environmental signals, cellular redox homeostasis, and circadian clocks. In this study, the multiple scales of coupled mechanisms provided a system-level understanding of several modules and network communities that are interlinked by hub regions mediating phytohormones synergy and signaling interdependency. Some of the enzymes and enzyme-coding genes or regulating TFs in the EAPCN model have been validated by physiological and genetic experimental studies (Additional file 3). From the view of modules and network communities, the highly interconnected enzymes in the EAPCN model and their enzyme-coding genes or regulating TFs would be the starting point for future experimental analysis. Such as, oxidoreductases (EC 1.14.13.-) and their enzyme-coding genes DWF4 (AT3G50660) and CYP79F2 (AT1G16400) or regulating TFs TCP1 (AT1G67260), PIL5 (AT2G20180). GH3 enzymes (EC: 6.3.-.-) and their enzymatic genes GH3.5 (AT4G27260) and DFL1 (AT5G54510) by modification of STY1 (AT3G51060). Hexosyltransferases (EC: 2.4.1.-) and enzymatic genes UGT78D2 (AT5G17050). Our results provide a broader view of phytohormone crosstalk in Arabidopsis and uncover potential key regulators that can be further analyzed in future studies.

## Additional files

Additional file 1: The detailed description for the bioinformatic analyses and methods. (DOCX 813 kb) Additional file 2: XML, XGMML, GML, SIF and NNF format of the identified models in the manuscript. (ZIP 164 kb) Additional file 3: Supplementary Information, section I. Existing physiological and genetic studies. Supplementary Information, section II. Transcription factors-based circular control units of endogenous phytohormone-biosynthetic enzymatic genes in EAPCN. Supplementary Information, section III. Distinct TFs mediating multiple target enzymatic gene of various phytohormone biosynthesis. (DOCX 516 kb)
