# Supplementary material for: The Arabidopsis phytohormone crosstalk network involves a consecutive metabolic route and circular control units of transcription factors that regulate enzyme-encoding genes
Source: BMC Syst Biol. 2016 Sep 2;10(1):87. doi: 10.1186/s12918-016-0333-9 (PMC5009710; doi:10.1186/s12918-016-0333-9)
Supplement: Additional file 1: — The detailed description for the bioinformatic analyses and methods. (DOCX 813 kb) [file 12918_2016_333_MOESM1_ESM.docx]

The detailed description for the bioinformatic analyses and methods

**Publicly available databases used in this study**

The *Arabidopsis* metabolic pathway database AraCyc 10.0 ([http://www.*Arabidopsis*.org/biocyc/](http://www.Arabidopsis.org/biocyc/)) contains biochemical pathways that represent *Arabidopsis* metabolism (Figure 1) [13]. Arabidopsis metabolic pathway database AraCyc 10. 0 (http://www.Arabidopsis.org/biocyc/), which is comprised 540 pathways, 7127 enzymes, 3418 reactions, 3323 compounds and 4225 citations, which is to analyze the global perspective of the active enzymes and metabolic routes .


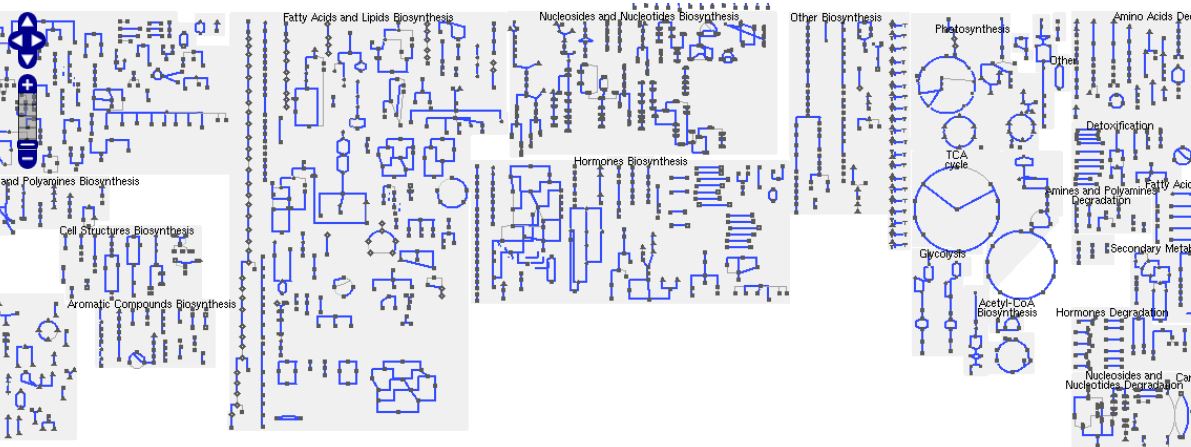


**Figure 1** Screenshot of the Metabolic Overview page of *Arabidopsis* metabolism from AraCyc 10.0 (http://www. Arabidopsis. org/biocyc/)[13].

**Genome-scale enzyme correlation network (GECN) model for *Arabidopsis***

As shown in figure 2, the GECN model contains active information (enzymes, reactions, compounds, and citations), the nodes represent the enzymes and edges represent two enzymes that interact with the same substrates. The detailed description and a reproducible code that was used to reconstructed the GECN model were depicted in our previously published GECN model [14].


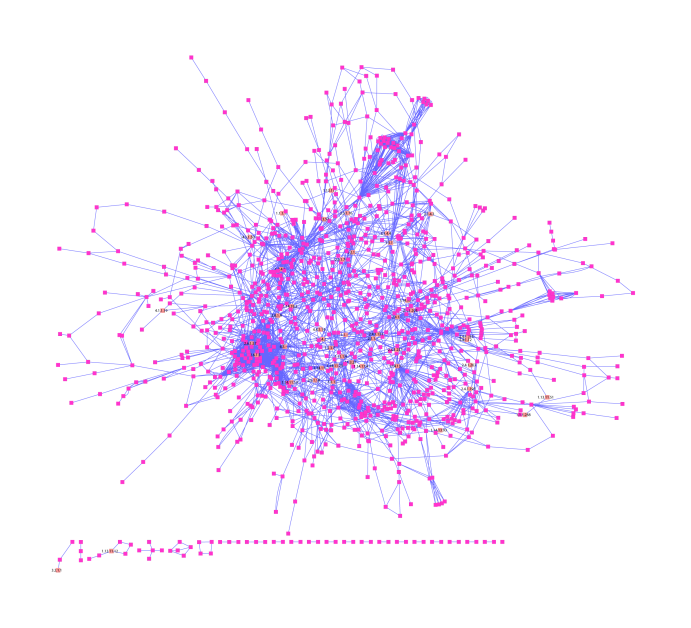


**Figure 2** Overview of the GECN model. Nodes represent enzymes, and edges indicate two enzymes that interact with the same substrates.

**Construction of the *Arabidopsis*** **phytohormone crosstalk network (****EAPCN) model**

The EAPCN model was constructed based on the GECN model as follows:

Step 1: Source files (TXT file) were downloaded from AraCyc 10.0 [13] and AHD2.0 [15].

Example of database from AraCyc 10.0:

| PID1 | 1.14.99.- |
| --- | --- |
| PREACTION_EQUATION | 1-18:2-2-16:1-monogalactosyldiacylglycerol -> 1-18:2-2-16:2-monogalactosyldiacylglycerol + 2 H+ glycolipid desaturation |
| PPATHWAY PPATHWAY_ID | PWY-762 |
| PREACTION_ID | RXN-8316 |
| PPROTEIN_NAME | &omega;-6 fatty acid desaturase |
| PGENE_ID | AT4G30950 |
| PANNOTATION | Acyl Lipid Metabolism |
| GENE_FAMILY | Family:Plastidial Oleate Desaturase (FAD6) |

Step 2: The TXT file of the *Arabidopsis* hormone information on eight major phytohormones, which includes pathways, enzymes, genes, reactions, compounds, and citations, were imported to an Oracle database platform.

Example of database

| GENE | AT1G60980 |
| --- | --- |
| RELATED_HORMONE | GIBBERELLIN |
| FUNCTION_CATEGORY | Hormone biosynthesis |
| PMID | 7604047; |
| PID1 | 1.14.11.- |
| PREACTION_EQUATION | GA24 + 2-oxoglutarate + oxygen -> GA36 + succinate + CO2 |
| PPATHWAY | gibberellin biosynthesis I (non C-3, non C-13 hydroxylation) |
| PPATHWAY_ID | PWY-5036 |
| PREACTION_ID | RXN-7591 |
| PPROTEIN_NAME | gibberellin 20-oxidase |
| PGENE_ID | AT1G60980 |
| PANNOTATION |  |

The detailed description and a reproducible code that was used to generate the data in the Oracle database platform

(select *

from HORMONE_database LEFT OUTER JOIN metabolic_ pathway_ database ON

RTRIM(LTRIM(HORMONE_database.gene))=RTRIM(LTRIM(metabolic_ pathway_ database.pgene_id)))

HORMONE_result : 431 genes ：

AT5G05580 AT1G15550 AT2G36800 AT4G00360 AT1G14130 AT1G06090 AT5G58660 AT5G12890

AT2G37760 AT4G35790 AT1G05680 AT4G37390 AT1G24100 AT1G80330 AT5G63600 AT3G61220

AT5G14200 AT1G06360 AT1G76130 AT3G53150 AT3G13610 AT1G77120 AT3G19010 AT1G70560

AT5G23190 AT5G45340 AT4G23600 AT1G35190 AT4G38190 AT3G46490 AT5G43935 AT2G30860

AT1G62380 AT3G49700 AT2G30870 AT5G13930 AT3G62760 AT2G29480 AT1G01090 AT5G16910

AT1G10360 AT3G02620 AT2G47120 AT1G55940 AT5G18930 AT5G64000 AT5G20960 AT3G46500

AT1G19570 AT2G19590 AT5G63980 AT1G78380 AT2G29440 AT3G44970 AT1G59670 AT4G21200

AT5G51810 AT1G30530 AT5G07200 AT2G27690 AT4G23340 AT3G15870 AT3G15850 AT4G36380

AT1G22380 AT3G25180 AT3G63520 AT1G22430 AT1G47990 AT2G31360 AT2G36970 AT5G55250

AT1G22440 AT1G78360 AT1G04420 AT4G25000 AT1G19630 AT1G06080 AT5G24760 AT1G22370

AT4G24010 AT5G59590 AT3G03190 AT1G59900 AT1G65670 AT5G17420 AT1G27140 AT2G29490

AT2G15490 AT4G32410 AT4G15260 AT2G29710 AT1G43800 AT5G20400 AT2G23210 AT3G03540

AT2G33100 AT2G43710 AT1G02950 AT1G28030 AT1G74470 AT1G60980 AT5G58860 AT3G50660

AT1G73880 AT2G43820 AT5G38010 AT3G04000 AT2G38050 AT3G63410 AT4G11830 AT5G23790

AT2G23170 AT5G04330 AT5G05730 AT4G37770 AT4G26250 AT1G23740 AT4G23590 AT5G43940

AT5G08640 AT1G55850 AT2G31790 AT2G25540 AT2G29740 AT5G63580 AT1G01480 AT1G78370

AT3G50210 AT2G47140 AT2G44800 AT3G46480 AT2G22590 AT5G09870 AT1G25083 AT1G07260

AT3G21780 AT4G19880 AT1G69920 AT1G77330 AT3G48610 AT3G02100 AT3G01420 AT1G75270

AT3G46660 AT2G37770 AT3G30180 AT5G37950 AT3G55360 AT3G14440 AT2G03220 AT2G29980

AT1G30040 AT1G22400 AT5G17050 AT2G34555 AT5G30500 AT1G78390 AT2G42010 AT4G22880

AT5G65550 AT4G38530 AT1G18870 AT4G16770 AT1G44090 AT2G27680 AT5G63620 AT1G24909

AT3G26770 AT2G02930 AT2G20610 AT5G07480 AT1G07250 AT3G16520 AT1G17170 AT3G24220

AT2G32530 AT5G44030 AT4G16765 AT4G19230 AT2G28880 AT1G17180 AT2G31570 AT2G23250

AT1G63460 AT5G41220 AT5G09290 AT5G17220 AT1G07230 AT1G02920 AT2G29690 AT4G02520

AT5G62480 AT5G14400 AT4G27260 AT3G53130 AT2G45970 AT1G28130 AT1G02400 AT1G14120

AT5G48000 AT2G31750 AT5G54510 AT1G10400 AT1G06120 AT1G53680 AT3G47190 AT5G45020

AT5G52570 AT4G26420 AT2G46370 AT1G64710 AT1G06000 AT1G67990 AT4G36220 AT1G06350

AT5G56300 AT3G45140 AT1G78270 AT3G53160 AT5G25370 AT1G30100 AT3G50740 AT3G49630

AT5G40390 AT4G00240 AT1G22340 AT5G20550 AT4G15320 AT2G02390 AT5G19440 AT3G02470

AT4G34710 AT2G36770 AT1G72520 AT3G02630 AT1G02050 AT4G08040 AT1G62960 AT4G18780

AT5G44000 AT3G49620 AT4G39350 AT1G49860 AT2G43350 AT1G74590 AT1G30120 AT5G41210

AT3G29260 AT3G43800 AT2G27150 AT4G15550 AT5G49690 AT2G26480 AT3G03530 AT3G51680

AT3G42960 AT1G69930 AT5G05860 AT4G18350 AT4G03070 AT4G27030 AT1G67730 AT2G36790

AT5G08380 AT5G05870 AT1G01600 AT5G54000 AT3G12120 AT3G29250 AT1G50960 AT1G59960

AT1G32780 AT2G43840 AT5G38970 AT1G04710 AT2G36780 AT2G32620 AT4G11840 AT3G21760

AT1G06100 AT5G65800 AT2G29750 AT2G28080 AT5G44990 AT3G60290 AT5G50850 AT4G15300

AT5G53580 AT1G24180 AT2G16500 AT1G27130 AT2G22810 AT3G16785 AT4G11600 AT1G24807

AT4G15280 AT1G25220 AT3G46670 AT5G16240 AT5G06580 AT3G30290 AT2G25080 AT1G25155

AT2G21770 AT2G29470 AT5G63990 AT5G16710 AT4G15393 AT2G32540 AT2G29420 AT4G30950

AT4G34138 AT1G59500 AT3G11170 AT3G13730 AT5G04660 AT2G36760 AT4G22110 AT1G16400

AT3G05630 AT2G33150 AT4G01970 AT5G54160 AT3G15730 AT1G54870 AT2G30140 AT1G69830

AT4G11850 AT4G25700 AT4G28410 AT3G22400 AT2G26870 AT1G12740 AT5G48880 AT2G30150

AT1G72970 AT1G06690 AT1G17420 AT4G23990 AT3G61510 AT1G49390 AT1G20510 AT5G36160

AT3G55870 AT1G51410 AT5G57890 AT2G24850 AT1G19550 AT5G15950 AT1G67560 AT5G53970

AT4G28420 AT1G02730 AT2G29450 AT1G32180 AT1G07240 AT2G34590 AT2G47730 AT4G14090

AT5G05690 AT3G02610 AT3G19270 AT1G52340 AT2G47130 AT1G10370 AT4G05530 AT2G44990

AT5G05170 AT2G32610 AT4G31870 AT3G26760 AT1G78490 AT5G41240 AT3G09270 AT1G05010

AT3G21800 AT3G63080 AT1G16540 AT1G80340 AT2G36750 AT1G62940 AT1G16410 AT4G25420

AT4G19170 AT3G21750 AT5G42250 AT1G22360 AT4G34135 AT1G78440 AT1G05675 AT2G24190

AT1G31800 AT5G51690 AT1G55180 AT3G22250 AT1G55020 AT1G05530 AT5G59580 AT1G52570

AT4G26200 AT1G23320 AT5G63590 AT1G78320 AT1G17190 AT4G15270 AT4G11280 AT1G78340

AT3G25570 AT1G74710 AT4G15396 AT1G12010 AT2G29730 AT2G36690 AT5G63595 AT2G23260

AT4G24000 AT5G64740 AT2G29460 AT1G02940 AT1G05560 AT4G03140 AT1G73680 AT4G15290

AT3G21790 AT3G03050 AT2G02380 AT5G16230 AT1G59700 AT2G29090 AT1G02930

select * from HORMONE_result

order by gene,related_hormone,pid1

Example of database

| GENE | AT4G27260 |
| --- | --- |
| PID1 | 6.3.-.- |
| RELATED_HORMONE | AUXIN |

HORMONE_result1: 342

result :

BRASSINOSTEROID 25 gene --- 14 enzyme

ABSCISIC ACID 80 gene --- 51 enzyme

AUXIN 43 gene --- 34 enzyme

CYTOKININ 33 gene --- 9 enzyme

ETHYLENE 45 gene --- 26 enzyme

GIBBERELLIN 44 gene --- 11 enzyme

JASMONIC ACID 54 gene --- 39 enzyme

SALICYLIC ACID 18 gene --- 16 enzyme

Plant Hormones Biosynthesis:

ABSCISIC ACID:

EC: 5.5.99.9 EC: 5.2.1.- EC: 1.13.11.51 EC: 1.1.1.288 EC: 1.2.3.14 EC: 2.4.1.263 EC: 1.14.13.93 EC:1.1.1.-

AUXIN:

EC: 2.4.1.- EC: 6.2.1.- EC: 1.3.-.- IBI3 EC: 4.2.-.- EC: 1.1.-.- IBI1 EC: 2.3.1.- EC: 3.1.2.-

BRASSINOSTEROID:

EC: 1.3.1.- EC: 1.1.1.- EC: 5.3.3.1 EC: 1.1.1.145 EC: 1.1.1.51 EC: 5.3.3.1 EC: 1.1.1.- EC: 1.3.1.- EC: 1.1.-.- EC: 1.14.-.- EC: 1.14.13.-

EC: 1.14.13.112 EC: 5.3.3.1 EC: 1.1.1.- EC: 1.3.1.- EC: 1.1.-.- EC: 1.14.-.- EC: 1.14.13.-

CYTOKININ:

EC: 2.4.1.203 EC: 2.4.1.215 EC: 2.4.1.- EC: 2.5.1.27 EC: 2.5.1.- EC: 3.2.2.- EC: 2.5.1.75

ETHYLENE:

EC:2.5.1.6 SAM-1 EC: 4.4.1.14 ACS2 EC: 1.14.17.4 ACO

GIBBERELLIN:

EC: 1.14.11.15 EC: 1.14.11.- EC: 1.14.11.12

JASMONIC ACID:

EC: 1.13.11.12 LOX6 EC: 4.2.1.92 CYP74A1 EC: 5.3.88.6 EC: 1.3.1.42 OPR3 EC: 6.2.1.- OPCL1 EC: 1.3.3.6 EC: 4.2.1.17 AIM1

EC:1.1.1.35 EC: 2.3.1.16 KAT2 EC: 1.3.3.6 EC: 4.2.1.17 EC: 1.1.1.35 EC: 2.3.1.16 KAT2 EC: 3.1.2.20 EC: 1.14.13.- EC: 2.8.2.-

SALICYLIC ACID:

4.3.1.24 -- phenylalanine ammonia-lyase 4.3.1.25 -- phenylalanine/tyrosine ammonia-lyase

Step 3: The enzymes in AraCyc were mapped to the GECN model, and a sub-interaction network was constructed on an Oracle database platform using structured query language (SQL).


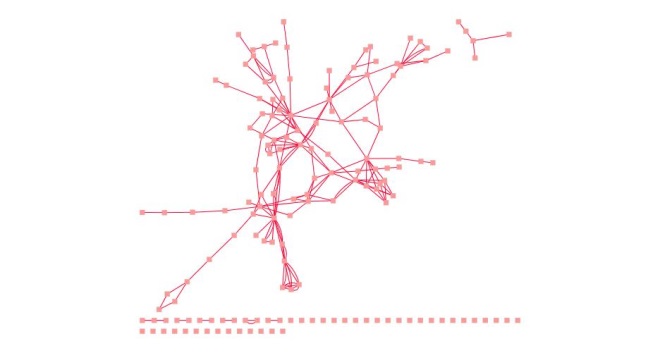


**Figure 3** Overview of the sub-interaction network

(1-abscisic acid, 2-auxin, 3-brassinosteroid, 4-cytokinin, 5-ethylene, 6-gibberellin, 7-jasmonic acid 8-salicylic acid)

PID11 beizhu RELATED_HORMONE1

1.-.-.- 2 AUXIN

1.1.-.- 23

1.1.1.- 13

1.1.1.1 58 ETHYLENE

1.1.1.145 3

1.1.1.219 3 BRASSINOSTEROID

1.1.1.22 5 ETHYLENE

1.1.1.27 1 ABSCISIC ACID

1.1.1.284 8 SALICYLIC ACID

1.1.1.288 13 ABSCISIC ACID

1.1.1.35 7 JASMONIC ACID

1.1.1.51 3

1.11.1.12 1 ABSCISIC ACID

1.11.1.6 1 ABSCISIC ACID

1.11.1.9 15 ABSCISIC ACID

1.13.11.- 178 ABSCISIC ACID

1.13.11.12 17 ABSCISIC ACID

1.13.11.51 125 ABSCISIC ACID

1.14.-.- 3 BRASSINOSTEROID

1.14.11.- 567 ETHYLENE

1.14.11.12 6 GIBBERELLIN

1.14.11.13 56 ETHYLENE

1.14.11.15 6 GIBBERELLIN

1.14.11.19 7 JASMONIC ACID

1.14.11.23 67 GIBBERELLIN

1.14.11.9 6 GIBBERELLIN

1.14.13.- 37 BRASSINOSTEROID

1.14.13.112 3 BRASSINOSTEROID

1.14.13.125 2 AUXIN

1.14.13.132 7 JASMONIC ACID

1.14.13.78 6 GIBBERELLIN

1.14.13.79 6 GIBBERELLIN

1.14.13.90 1 ABSCISIC ACID

1.14.13.93 135 BRASSINOSTEROID

1.14.17.4 5 ETHYLENE

1.14.19.2 78 JASMONIC ACID

1.14.99.- 157 ABSCISIC ACID

1.14.99.33 7 JASMONIC ACID

1.2.1.- 3 BRASSINOSTEROID

1.2.1.28 1 ABSCISIC ACID

1.2.1.3 1 ABSCISIC ACID

1.2.1.31 1 ABSCISIC ACID

1.2.1.41 1 ABSCISIC ACID

1.2.3.14 1 ABSCISIC ACID

1.2.3.7 12 ABSCISIC ACID

1.2.4.1 25 AUXIN

1.3.-.- 2

1.3.1.- 13 ABSCISIC ACID

1.3.1.33 5 ETHYLENE

1.3.1.42 7

1.3.3.6 7

1.5.3.14 8 SALICYLIC ACID

1.5.3.17 8 SALICYLIC ACID

1.5.99.12 4 CYTOKININ

1.8.5.1 7 JASMONIC ACID

2.1.1.- 26 AUXIN

2.1.2.1 8 SALICYLIC ACID

2.10.1.1 2 AUXIN

2.2.1.7 1 ABSCISIC ACID

2.3.1.- 2

2.3.1.16 17 ABSCISIC ACID

2.3.1.20 1 ABSCISIC ACID

2.3.1.74 27 AUXIN

2.4.1.- 12348 ABSCISIC ACID

2.4.1.115 1 ABSCISIC ACID

2.4.1.12 157 ABSCISIC ACID

2.4.1.121 128 ABSCISIC ACID

2.4.1.195 18 ABSCISIC ACID

2.4.1.203 34 BRASSINOSTEROID

2.4.1.215 34 BRASSINOSTEROID

2.4.1.237 1238 ABSCISIC ACID

2.4.1.263 128 ABSCISIC ACID

2.4.1.34 8 SALICYLIC ACID

2.4.1.43 5 ETHYLENE

2.4.1.46 4 CYTOKININ

2.4.1.91 1 ABSCISIC ACID

2.5.1.- 4 CYTOKININ

2.5.1.16 2 AUXIN

2.5.1.18 578 ETHYLENE

2.5.1.22 2 AUXIN

2.5.1.27 4 CYTOKININ

2.5.1.29 1 ABSCISIC ACID

2.5.1.6 5 ETHYLENE

2.5.1.75 4 CYTOKININ

2.5.1.78 7 JASMONIC ACID

2.5.1.9 7 JASMONIC ACID

2.6.1.- 2 AUXIN

2.6.1.1 5 ETHYLENE

2.6.1.2 2 AUXIN

2.6.1.27 2 AUXIN

2.6.1.42 2 AUXIN

2.6.1.5 127 ABSCISIC ACID

2.6.1.57 25 AUXIN

2.6.1.58 2 AUXIN

2.7.1.33 5 ETHYLENE

2.7.2.11 1 ABSCISIC ACID

2.7.4.6 2 AUXIN

2.7.7.41 5 ETHYLENE

2.7.7.69 7 JASMONIC ACID

2.7.7.75 2 AUXIN

2.7.8.11 1 ABSCISIC ACID

2.7.9.1 5 ETHYLENE

2.8.1.12 2 AUXIN

2.8.1.9 12 ABSCISIC ACID

2.8.2.- 7

3.1.1.14 7 JASMONIC ACID

3.1.1.32 7 JASMONIC ACID

3.1.1.4 2 AUXIN

3.1.2.- 2

3.1.2.20 7

3.1.2.4 2 AUXIN

3.1.3.2 7 JASMONIC ACID

3.1.3.2 7 JASMONIC ACID

3.1.3.4 1 ABSCISIC ACID

3.1.3.56 1 ABSCISIC ACID

3.1.3.57 12 ABSCISIC ACID

3.1.3.81 1 ABSCISIC ACID

3.1.4.11 1 ABSCISIC ACID

3.1.4.3 23 AUXIN

3.1.4.4 127 ABSCISIC ACID

3.2.1.1 16 ABSCISIC ACID

3.2.1.21 1 ABSCISIC ACID

3.2.2.- 4

3.5.1.19 1 ABSCISIC ACID

3.5.2.17 3 BRASSINOSTEROID

3.6.1.22 8 SALICYLIC ACID

4.1.1.19 17 ABSCISIC ACID

4.1.1.49 5 ETHYLENE

4.1.1.50 24 AUXIN

4.1.2.13 5 ETHYLENE

4.1.3.27 257 AUXIN

4.1.99.5 1 ABSCISIC ACID

4.2.-.- 2

4.2.1.1 5 ETHYLENE

4.2.1.17 7 JASMONIC ACID

4.2.1.92 7 JASMONIC ACID

4.2.1.92 7

4.2.3.- 7 JASMONIC ACID

4.2.3.14 7 JASMONIC ACID

4.2.3.15 7 JASMONIC ACID

4.2.3.16 7 JASMONIC ACID

4.3.1.24 8

4.3.1.25 8

4.4.1.14 2457 AUXIN

4.4.1.8 7 ABSCISIC ACID

5.2.1.- 1

5.3.3.1 3

5.3.88.6 7

5.3.99.6 7 JASMONIC ACID

5.3.99.9 1 ABSCISIC ACID

5.4.4.2 58 ETHYLENE

5.5.1.13 6 GIBBERELLIN

5.5.99.9 1

6.1.1.7 1 ABSCISIC ACID

6.2.1.- 27 JASMONIC ACID

6.2.1.12 7 JASMONIC ACID

6.2.1.3 1 ABSCISIC ACID

6.3.-.- 127 ABSCISIC ACID

6.3.2.2 7 JASMONIC ACID

6.3.2.6 2 AUXIN

6.3.4.14 4 CYTOKININ

6.4.1.2 4 CYTOKININ

6.6.1.1 1 ABSCISIC ACID

Step 4: To infer antagonistic crosstalk between phytohormones, we included structural connectivity architecture at the biosynthesis level as another feature in the EAPCN model (Figure 4). The EAPCN model provides a new platform for analyzing how multiple scales of collaborative mechanisms work from a global perspective.

(1-abscisic acid, 2-auxin, 3-brassinosteroid, 4-cytokinin, 5-ethylene, 6-gibberellin, 7-jasmonic acid 8-salicylic acid)

1.1.-.- 23

1.1.1.- 13

1.1.1.1 58

1.1.1.288 13

1.11.1.9 15

1.13.11.- 178

1.13.11.12 17

1.13.11.51 125

1.14.11.- 567

1.14.11.13 56

1.14.11.23 67

1.14.13.- 37

1.14.13.93 135

1.14.17.4 5

1.14.19.2 78

1.14.99.- 157

1.2.3.7 12

1.2.4.1 25

1.3.1.- 13

2.1.1.- 26

2.3.1.16 17

2.3.1.74 27

2.4.1.- 12348

2.4.1.12 157

2.4.1.121 128

2.4.1.195 18

2.4.1.203 34

2.4.1.215 34

2.4.1.237 1238

2.4.1.263 128

2.5.1.18 578

2.6.1.5 127

2.6.1.57 25

2.8.1.9 12

3.1.3.57 12

3.1.4.3 23

3.1.4.4 127

3.2.1.1 16

4.1.1.19 17

4.1.1.50 24

4.1.3.27 257

4.4.1.14 2457

5.4.4.2 58

6.2.1.- 27

6.3.-.- 127


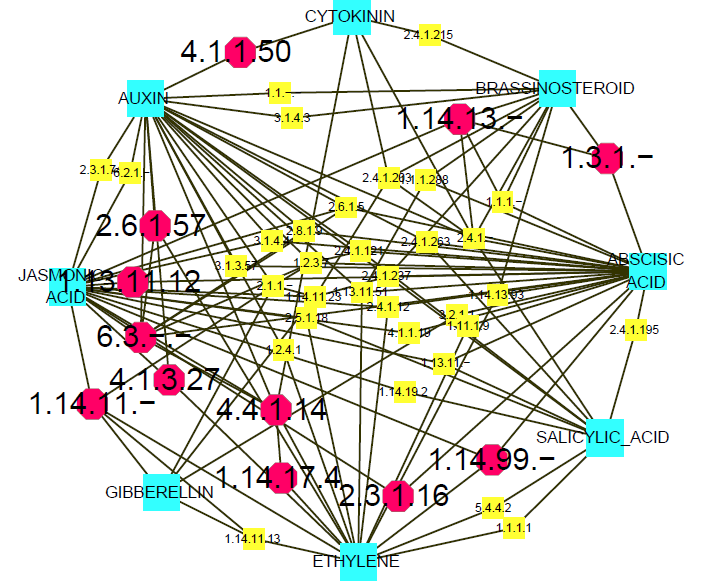


**Figure 4**: Structural connectivity of the enzyme-based Arabidopsis phytohormones crosstalk network (EAPCN) at the biosynthesis level, and attached as supporting information (Additional_files2.zip) in SBML format (*.xml and *.xgmml)

ABSCISIC ACID (PP) 1.1.1.-

ABSCISIC ACID (PP) 1.1.1.288

ABSCISIC ACID (PP) 1.11.1.9

ABSCISIC ACID (PP) 1.13.11.-

ABSCISIC ACID (PP) 1.13.11.12

ABSCISIC ACID (PP) 1.13.11.51

ABSCISIC ACID (PP) 1.14.13.93

ABSCISIC ACID (PP) 1.14.99.-

ABSCISIC ACID (PP) 1.2.3.7

ABSCISIC ACID (PP) 1.3.1.-

ABSCISIC ACID (PP) 2.3.1.16

ABSCISIC ACID (PP) 2.4.1.-

ABSCISIC ACID (PP) 2.4.1.12

ABSCISIC ACID (PP) 2.4.1.121

ABSCISIC ACID (PP) 2.4.1.195

ABSCISIC ACID (PP) 2.4.1.237

ABSCISIC ACID (PP) 2.4.1.263

ABSCISIC ACID (PP) 2.6.1.5

ABSCISIC ACID (PP) 2.8.1.9

ABSCISIC ACID (PP) 3.1.3.57

ABSCISIC ACID (PP) 3.1.4.4

ABSCISIC ACID (PP) 3.2.1.1

ABSCISIC ACID (PP) 4.1.1.19

ABSCISIC ACID (PP) 6.3.-.-

AUXIN (PP) 1.1.-.-

AUXIN (PP) 1.13.11.51

AUXIN (PP) 1.2.3.7

AUXIN (PP) 1.2.4.1

AUXIN (PP) 2.1.1.-

AUXIN (PP) 2.3.1.74

AUXIN (PP) 2.4.1.-

AUXIN (PP) 2.4.1.121

AUXIN (PP) 2.4.1.237

AUXIN (PP) 2.4.1.263

AUXIN (PP) 2.6.1.5

AUXIN (PP) 2.6.1.57

AUXIN (PP) 2.8.1.9

AUXIN (PP) 3.1.3.57

AUXIN (PP) 3.1.4.3

AUXIN (PP) 3.1.4.4

AUXIN (PP) 4.1.1.50

AUXIN (PP) 4.1.3.27

AUXIN (PP) 4.4.1.14

AUXIN (PP) 6.2.1.-

AUXIN (PP) 6.3.-.-

BRASSINOSTEROID (PP) 1.1.-.-

BRASSINOSTEROID (PP) 1.1.1.-

BRASSINOSTEROID (PP) 1.1.1.288

BRASSINOSTEROID (PP) 1.14.13.-

BRASSINOSTEROID (PP) 1.14.13.93

BRASSINOSTEROID (PP) 1.3.1.-

BRASSINOSTEROID (PP) 2.4.1.-

BRASSINOSTEROID (PP) 2.4.1.203

BRASSINOSTEROID (PP) 2.4.1.215

BRASSINOSTEROID (PP) 2.4.1.237

BRASSINOSTEROID (PP) 3.1.4.3

CYTOKININ (PP) 2.4.1.-

CYTOKININ (PP) 2.4.1.203

CYTOKININ (PP) 2.4.1.215

CYTOKININ (PP) 4.1.1.50

CYTOKININ (PP) 4.4.1.14

ETHYLENE (PP) 1.1.1.1

ETHYLENE (PP) 1.11.1.9

ETHYLENE (PP) 1.13.11.51

ETHYLENE (PP) 1.14.11.-

ETHYLENE (PP) 1.14.11.13

ETHYLENE (PP) 1.14.13.93

ETHYLENE (PP) 1.14.17.4

ETHYLENE (PP) 1.14.99.-

ETHYLENE (PP) 1.2.4.1

ETHYLENE (PP) 2.4.1.12

ETHYLENE (PP) 2.5.1.18

ETHYLENE (PP) 2.6.1.57

ETHYLENE (PP) 4.1.3.27

ETHYLENE (PP) 4.4.1.14

ETHYLENE (PP) 5.4.4.2

GIBBERELLIN (PP) 1.14.11.-

GIBBERELLIN (PP) 1.14.11.13

GIBBERELLIN (PP) 1.14.11.23

GIBBERELLIN (PP) 2.1.1.-

GIBBERELLIN (PP) 3.2.1.1

JASMONIC_ ACID (PP) 1.13.11.-

JASMONIC_ ACID (PP) 1.13.11.12

JASMONIC_ ACID (PP) 1.14.11.-

JASMONIC_ ACID (PP) 1.14.11.23

JASMONIC_ ACID (PP) 1.14.13.-

JASMONIC_ ACID (PP) 1.14.19.2

JASMONIC_ ACID (PP) 1.14.99.-

JASMONIC_ ACID (PP) 2.3.1.16

JASMONIC_ ACID (PP) 2.3.1.74

JASMONIC_ ACID (PP) 2.4.1.12

JASMONIC_ ACID (PP) 2.5.1.18

JASMONIC_ ACID (PP) 2.6.1.5

JASMONIC_ ACID (PP) 3.1.4.4

JASMONIC_ ACID (PP) 4.1.1.19

JASMONIC_ ACID (PP) 4.1.3.27

JASMONIC_ ACID (PP) 4.4.1.14

JASMONIC_ ACID (PP) 6.2.1.-

JASMONIC_ ACID (PP) 6.3.-.-

SALICYLIC_ACID (PP) 1.1.1.1

SALICYLIC_ACID (PP) 1.13.11.-

SALICYLIC_ACID (PP) 1.14.19.2

SALICYLIC_ACID (PP) 2.4.1.-

SALICYLIC_ACID (PP) 2.4.1.121

SALICYLIC_ACID (PP) 2.4.1.195

SALICYLIC_ACID (PP) 2.4.1.237

SALICYLIC_ACID (PP) 2.4.1.263

SALICYLIC_ACID (PP) 2.5.1.18

SALICYLIC_ACID (PP) 5.4.4.2

1.13.11.51 (PP) 1.1.1.288

2.3.1.74 (PP) 6.2.1.-

1.1.1.- (PP) 1.14.13.-

1.3.1.- (PP) 1.14.13.-

2.4.1.- (PP) 1.14.13.-

2.4.1.203 (PP) 2.4.1.-

1.14.11.23 (PP) 2.4.1.203

1.14.11.23 (PP) 2.4.1.237

1.14.11.23 (PP) 2.1.1.-

1.14.11.- (PP) 2.1.1.-

1.14.11.13 (PP) 1.14.11.-

1.2.3.7 (PP) 2.1.1.-

1.2.3.7 (PP) 2.4.1.121

1.2.3.7 (PP) 6.3.-.-

2.8.1.9 (PP) 6.3.-.-

6.3.-.- (PP) 2.6.1.5

6.3.-.- (PP) 2.6.1.57

6.3.-.- (PP) 4.4.1.14

4.4.1.14 (PP) 1.14.17.4

***Arabidopsis* transcription factors (TFs) interactome network**

To characterize the TFs that regulate the enzyme-encoding genes in the EAPCN model, we constructed a TF interactome network using the *Arabidopsis* TF data in PlnTFDB [19]. In the TFs interactome network, the nodes represent the TFs and the edges indicate two TFs that have the same target gene. The interactome network was constructed on an Oracle database platform using SQL and visualized using Cytoscape software [18]. Results was attached as supporting information (Additional_files2.zip) in SBML format (*.xml and *.xgmml).

Example of database (1431 records):

| TF_ID | AT1G01060 |
| --- | --- |
| TARGET_ID | AT5G61380 |
| TF_ALIAS | LHY |
| TARGET_ALIAS | TOC1 |

Circular control units of transcription factors (TFs) that regulate genes encoding enzymes in the EAPCN.

CORR_EC CORR_LIXI PGENE_ID TF_ID TARGET_ID TF_ALIAS TARGET_ALIAS

1.14.11.- 567 AT1G80340 AT2G20180 AT1G80340 PIL5 GA3OX2

6.3.-.- 127 AT4G27260 AT1G77850 AT4G27260 ARF17 WES1

1.14.11.- 567 AT1G15550 AT3G26790 AT1G15550 FUS3 GA3OX1

6.3.-.- 127 AT1G28130 AT5G37020 AT1G28130 ARF8 GH3.17

1.14.11.- 567 AT4G25420 AT3G61890 AT4G25420 ATHB-12 GA20OX1

6.3.-.- 127 AT4G37390 AT3G51060 AT4G37390 STY1 BRU6

2.1.1.- 26 AT5G54160 AT1G79180 AT5G54160 MYB63 ATOMT1

1.14.11.- 567 AT4G22880 AT4G34990 AT4G22880 MYB32 LDOX

1.13.11.12 17 AT1G17420 AT1G27730 AT1G17420 STZ LOX3

1.14.11.23 67 AT4G25420 AT1G14920 AT4G25420 GAI GA20OX1

4.4.1.14 2457 AT2G22810 AT3G59060 AT2G22810 PIL6 ACS4

5.4.4.2 58 AT1G74710 AT1G10170 AT1G74710 ATNFXL1 EDS16

1.14.11.13 56 AT1G02400 AT5G13790 AT1G02400 AGL15 GA2OX6

1.14.11.13 56 AT1G30040 AT2G20180 AT1G30040 PIL5 GA2OX2

1.14.11.- 567 AT1G15550 AT1G14920 AT1G15550 GAI GA3OX1

1.14.11.- 567 AT1G15550 AT4G36930 AT1G15550 SPT GA3OX1

1.14.13.- 37 AT1G16410 AT5G07690 AT1G16410 MYB29 CYP79F1

6.3.-.- 127 AT5G54510 AT5G37020 AT5G54510 ARF8 DFL1

2.4.1.- 12348 AT2G03220 AT3G18990 AT2G03220 VRN1 FT1

1.14.13.- 37 AT1G16400 AT5G07690 AT1G16400 MYB29 CYP79F2

1.14.99.- 157 AT1G78390 AT2G20180 AT1G78390 PIL5 NCED9

1.13.11.12 17 AT3G45140 AT5G01900 AT3G45140 WRKY62 LOX2

1.14.11.23 67 AT4G25420 AT4G08150 AT4G25420 KNAT1 GA20OX1

1.14.11.23 67 AT4G22880 AT5G41315 AT4G22880 GL3 LDOX

2.4.1.12 157 AT5G17420 AT3G13890 AT5G17420 MYB26 IRX3

6.3.-.- 127 AT4G27260 AT3G51060 AT4G27260 STY1 WES1

1.14.11.- 567 AT1G50960 AT1G12610 AT1G50960 DDF1 GA2OX7

1.14.11.23 67 AT4G22880 AT4G34990 AT4G22880 MYB32 LDOX

2.5.1.18 578 AT2G47730 AT3G50410 AT2G47730 OBP1 GSTF8

1.14.11.13 56 AT1G47990 AT1G62360 AT1G47990 BUM1 GA2OX4

1.14.11.- 567 AT1G02400 AT4G25490 AT1G02400 CBF1 GA2OX6

1.14.11.- 567 AT4G25420 AT4G08150 AT4G25420 KNAT1 GA20OX1

6.3.-.- 127 AT5G54510 AT1G77850 AT5G54510 ARF17 DFL1

1.14.11.- 567 AT4G22880 AT1G63650 AT4G22880 EGL3 LDOX

1.14.11.23 67 AT4G25420 AT3G61890 AT4G25420 ATHB-12 GA20OX1

3.1.4.4 127 AT3G16785 AT1G79840 AT3G16785 GL2 PLDP1

2.3.1.74 27 AT5G13930 AT3G17609 AT5G13930 HYH TT4

2.5.1.18 578 AT5G17220 AT1G56650 AT5G17220 MYB75 GSTF12

1.14.11.- 567 AT1G80340 AT3G26790 AT1G80340 FUS3 GA3OX2

1.14.11.- 567 AT1G15550 AT3G61850 AT1G15550 DAG1 GA3OX1

1.14.13.- 37 AT3G50660 AT2G20180 AT3G50660 PIL5 DWF4

1.14.11.- 567 AT1G02400 AT5G13790 AT1G02400 AGL15 GA2OX6

1.14.11.- 567 AT4G25420 AT1G14920 AT4G25420 GAI GA20OX1

6.3.-.- 127 AT5G54510 AT3G51060 AT5G54510 STY1 DFL1

1.14.11.- 567 AT4G22880 AT5G35550 AT4G22880 TT2 LDOX

4.4.1.14 2457 AT4G37770 AT5G62000 AT4G37770 ARF2 ACS8

1.13.11.12 17 AT3G45140 AT1G52890 AT3G45140 ANAC019 LOX2

1.1.1.1 58 AT1G77120 AT1G45249 AT1G77120 ABF2 ADH1

1.13.11.12 17 AT1G17420 AT1G62300 AT1G17420 WRKY6 LOX3

1.14.11.23 67 AT4G22880 AT1G09530 AT4G22880 PIF3 LDOX

4.4.1.14 2457 AT1G01480 AT1G19850 AT1G01480 MP ACS2

5.4.4.2 58 AT1G74710 AT3G28910 AT1G74710 MYB30 EDS16

1.14.13.93 135 AT3G50660 AT1G75080 AT3G50660 BZR1 DWF4

1.13.11.51 125 AT3G24220 AT2G20180 AT3G24220 PIL5 NCED6

1.14.11.13 56 AT2G34555 AT4G25490 AT2G34555 CBF1 GA2OX3

2.5.1.18 578 AT1G02930 AT1G52890 AT1G02930 ANAC019 GSTF6

6.3.-.- 127 AT4G27260 AT5G20730 AT4G27260 ARF7 WES1

1.13.11.12 17 AT3G45140 AT4G31550 AT3G45140 WRKY11 LOX2

1.14.11.23 67 AT4G22880 AT1G63650 AT4G22880 EGL3 LDOX

4.4.1.14 2457 AT1G01480 AT5G62000 AT1G01480 ARF2 ACS2

2.6.1.5 127 AT2G24850 AT1G32640 AT2G24850 MYC2 TAT3

1.14.13.93 135 AT3G50660 AT2G20180 AT3G50660 PIL5 DWF4

1.14.11.13 56 AT1G30040 AT1G62360 AT1G30040 BUM1 GA2OX2

1.14.11.- 567 AT1G80340 AT1G28300 AT1G80340 LEC2 GA3OX2

6.3.-.- 127 AT1G59500 AT3G51060 AT1G59500 STY1 GH3.4

1.14.13.- 37 AT3G50660 AT1G75080 AT3G50660 BZR1 DWF4

1.14.11.- 567 AT1G30040 AT2G20180 AT1G30040 PIL5 GA2OX2

1.14.11.- 567 AT2G34555 AT4G25490 AT2G34555 CBF1 GA2OX3

5.4.4.2 58 AT1G74710 AT3G20770 AT1G74710 EIN3 EDS16

2.5.1.18 578 AT1G19570 AT5G23000 AT1G19570 MYB37 DHAR1

1.14.11.- 567 AT1G15550 AT2G01570 AT1G15550 RGA1 GA3OX1

1.14.11.- 567 AT4G25420 AT2G01570 AT4G25420 RGA1 GA20OX1

1.1.1.1 58 AT1G77120 AT2G47190 AT1G77120 MYB2 ADH1

1.13.11.12 17 AT1G17420 AT1G74930 AT1G17420 ORA47 LOX3

1.14.11.23 67 AT5G08640 AT2G47460 AT5G08640 MYB12 FLS1

1.14.13.93 135 AT3G50660 AT1G67260 AT3G50660 TCP1 DWF4

2.4.1.12 157 AT5G17420 AT1G71930 AT5G17420 VND7 IRX3

1.14.13.- 37 AT3G50660 AT1G67260 AT3G50660 TCP1 DWF4

1.14.13.- 37 AT1G16410 AT1G73730 AT1G16410 EIL3 CYP79F1

1.14.11.- 567 AT1G30040 AT1G62360 AT1G30040 BUM1 GA2OX2

2.4.1.- 12348 AT5G17050 AT1G56650 AT5G17050 MYB75 UGT78D2

4.1.3.27 257 AT5G05730 AT5G60890 AT5G05730 MYB34 AT5G05730

1.14.11.- 567 AT4G22880 AT5G41315 AT4G22880 GL3 LDOX

1.13.11.51 125 AT1G78390 AT2G20180 AT1G78390 PIL5 NCED9

2.3.1.74 27 AT5G13930 AT1G56650 AT5G13930 MYB75 TT4

1.14.11.13 56 AT1G02400 AT4G25490 AT1G02400 CBF1 GA2OX6

1.14.11.- 567 AT1G15550 AT4G18960 AT1G15550 AG GA3OX1

1.14.11.- 567 AT1G15550 AT4G25490 AT1G15550 CBF1 GA3OX1

1.14.11.- 567 AT4G25420 AT1G62360 AT4G25420 BUM1 GA20OX1

6.3.-.- 127 AT5G54510 AT2G20180 AT5G54510 PIL5 DFL1

2.4.1.- 12348 AT5G17050 AT2G16910 AT5G17050 AMS AT5G17050

1.14.11.- 567 AT4G22880 AT1G09530 AT4G22880 PIF3 LDOX

4.4.1.14 2457 AT4G37770 AT1G19850 AT4G37770 MP ACS8

1.14.11.23 67 AT4G22880 AT5G11260 AT4G22880 HY5 LDOX

4.4.1.14 2457 AT4G11280 AT1G19850 AT4G11280 MP ACS6

2.6.1.5 127 AT2G20610 AT5G60890 AT2G20610 MYB34 SUR1

1.14.11.13 56 AT1G50960 AT1G12610 AT1G50960 DDF1 GA2OX7

2.5.1.18 578 AT2G47730 AT1G69120 AT2G47730 AP1 GSTF8

1.14.11.- 567 AT1G15550 AT2G20180 AT1G15550 PIL5 GA3OX1

4.4.1.14 2457 AT4G37770 AT3G59060 AT4G37770 PIL6 ACS8

1.14.11.23 67 AT4G22880 AT5G35550 AT4G22880 TT2 LDOX

4.4.1.14 2457 AT2G22810 AT1G28300 AT2G22810 LEC2 ACS4

2.3.1.74 27 AT5G13930 AT2G43010 AT5G13930 PIF4 TT4

2.3.1.74 27 AT5G13930 AT1G02340 AT5G13930 HFR1 TT4

2.3.1.74 27 AT5G13930 AT4G38620 AT5G13930 MYB4 TT4

1.14.17.4 5 AT2G19590 AT3G01470 AT2G19590 ATHB-1 ATACO1

1.14.11.- 567 AT4G22880 AT5G11260 AT4G22880 HY5 LDOX

2.1.1.- 26 AT1G67990 AT2G16910 AT1G67990 AMS TSM1

1.13.11.12 17 AT3G45140 AT3G15500 AT3G45140 ANAC055 LOX2

1.13.11.12 17 AT1G17420 AT1G32640 AT1G17420 MYC2 LOX3

1.14.11.23 67 AT5G08640 AT5G41315 AT5G08640 GL3 FLS1

1.14.11.23 67 AT4G25420 AT2G01570 AT4G25420 RGA1 GA20OX1

4.4.1.14 2457 AT4G11280 AT5G62000 AT4G11280 ARF2 ACS6

5.4.4.2 58 AT1G74710 AT2G27050 AT1G74710 EIL1 EDS16

1.14.17.4 5 AT1G62380 AT3G20770 AT1G62380 EIN3 ATACO2

2.5.1.18 578 AT2G47730 AT3G16770 AT2G47730 RAP2.3 GSTF8

6.3.-.- 127 AT4G37390 AT5G20730 AT4G37390 ARF7 BRU6

1.14.13.- 37 AT1G16400 AT1G73730 AT1G16400 EIL3 CYP79F2

1.14.11.- 567 AT1G47990 AT1G62360 AT1G47990 BUM1 GA2OX4

2.4.1.12 157 AT4G18780 AT3G13890 AT4G18780 MYB26 IRX1

2.3.1.74 27 AT5G13930 AT2G47460 AT5G13930 MYB12 TT4

2.3.1.74 27 AT5G13930 AT1G09530 AT5G13930 PIF3 TT4

2.3.1.74 27 AT5G13930 AT5G11260 AT5G13930 HY5 TT4

2.3.1.74 27 AT5G13930 AT5G41315 AT5G13930 GL3 TT4

2.4.1.121 128 AT4G14090 AT1G56650 AT4G14090 MYB75 AT4G14090

1.14.13.93 135 AT5G05690 AT1G75080 AT5G05690 BZR1 CPD

1.14.13.93 135 AT2G29090 AT2G20180 AT2G29090 PIL5 CYP707A2

2.1.1.- 26 AT5G54160 AT1G16490 AT5G54160 MYB58 ATOMT1

1.13.11.12 17 AT3G45140 AT1G32640 AT3G45140 MYC2 LOX2

1.13.11.12 17 AT3G45140 AT2G24570 AT3G45140 WRKY17 LOX2

1.1.1.1 58 AT1G77120 AT1G32640 AT1G77120 MYC2 ADH1

1.13.11.12 17 AT1G17420 AT2G03340 AT1G17420 WRKY3 LOX3

1.14.11.23 67 AT5G08640 AT1G63650 AT5G08640 EGL3 FLS1

1.14.11.23 67 AT4G25420 AT1G62360 AT4G25420 BUM1 GA20OX1

2.3.1.74 27 AT5G13930 AT1G63650 AT5G13930 EGL3 TT4

1.2.3.7 12 AT5G20960 AT2G20180 AT5G20960 PIL5 AAO1

1.11.1.9 15 AT5G17220 AT1G56650 AT5G17220 MYB75 GSTF12

1.14.11.- 567 AT1G80340 AT4G36930 AT1G80340 SPT GA3OX2

6.3.-.- 127 AT2G23170 AT3G51060 AT2G23170 STY1 GH3.3

1.1.1.1 58 AT1G77120 AT1G35515 AT1G77120 HOS10 ADH1

1.1.1.1 58 AT1G77120 AT1G53910 AT1G77120 RAP2.12 ADH1

1.1.1.1 58 AT1G77120 AT4G25470 AT1G77120 CBF2 ADH1

2.6.1.5 127 AT2G20610 AT5G61420 AT2G20610 MYB28 SUR1

1.14.17.4 5 AT2G19590 AT3G20770 AT2G19590 EIN3 ATACO1


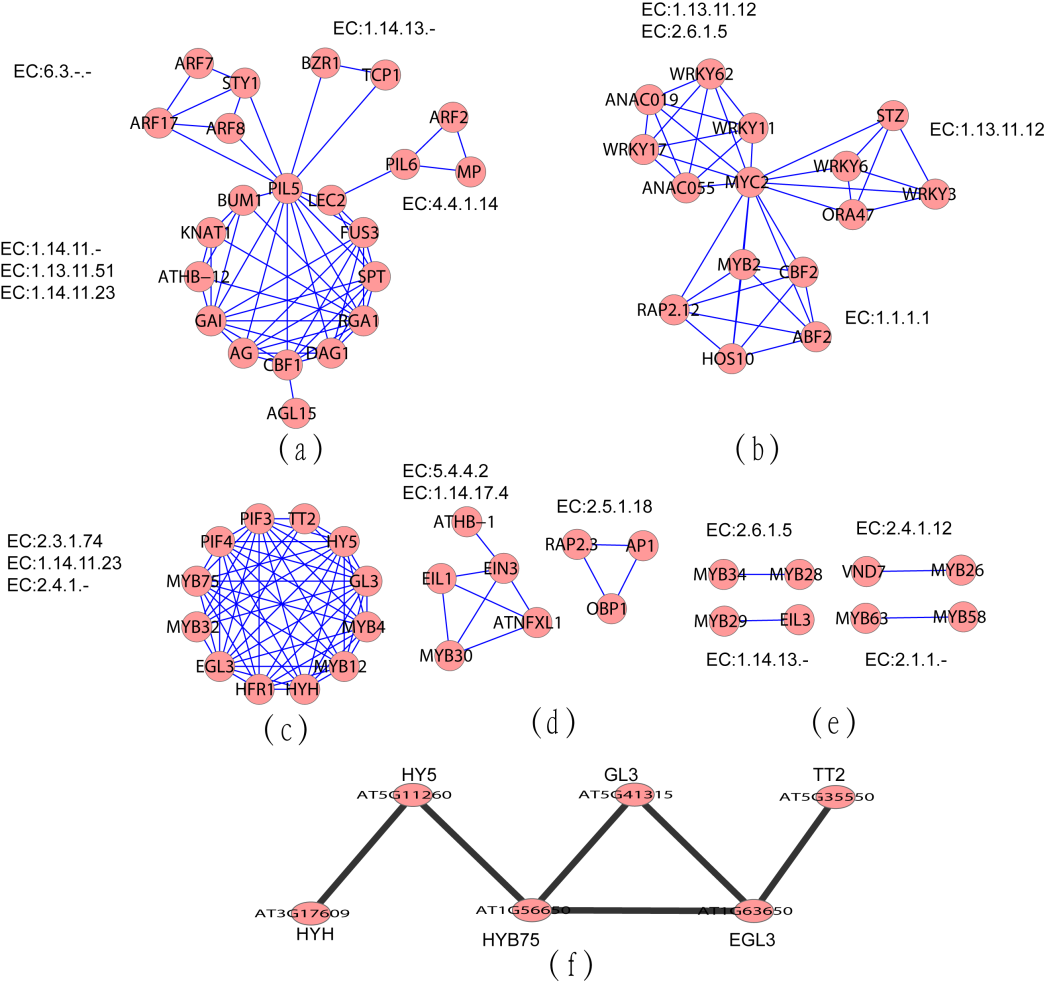


**Figure 5** Circular control units of transcription factors (TFs) that regulate genes encoding enzymes in the EAPCN. Nodes represent the TFs, edges indicate two TFs that have the same target gene. (a)–(c) Circular control units of TFs that regulate genes that encode enzymes in EAPCN (see Additional file 1, section II for details), (f) Consecutive protein–protein interaction route in circular control unit C.

HFR1 pp TT4

PIF3 pp LDOX

PIF3 pp TT4

ATNFXL1 pp EDS16

DDF1 pp GA2OX7

GAI pp GA20OX1

GAI pp GA3OX1

MYB58 pp ATOMT1

MP pp ACS2

MP pp ACS6

MP pp ACS8

STZ pp LOX3

LEC2 pp ACS4

LEC2 pp GA3OX2

MYC2 pp ADH1

MYC2 pp LOX2

MYC2 pp LOX3

MYC2 pp TAT3

HOS10 pp ADH1

ABF2 pp ADH1

ANAC019 pp GSTF6

ANAC019 pp LOX2

RAP2.12 pp ADH1

MYB75 pp AT4G14090

MYB75 pp GSTF12

MYB75 pp TT4

MYB75 pp UGT78D2

WRKY6 pp LOX3

BUM1 pp GA20OX1

BUM1 pp GA2OX2

BUM1 pp GA2OX4

EGL3 pp FLS1

EGL3 pp LDOX

EGL3 pp TT4

TCP1 pp DWF4

AP1 pp GSTF8

VND7 pp IRX3

EIL3 pp CYP79F1

EIL3 pp CYP79F2

ORA47 pp LOX3

BZR1 pp CPD

BZR1 pp DWF4

ARF17 pp DFL1

ARF17 pp WES1

MYB63 pp ATOMT1

GL2 pp PLDP1

RGA1 pp GA20OX1

RGA1 pp GA3OX1

WRKY3 pp LOX3

AMS pp AT5G17050

AMS pp TSM1

PIL5 pp AAO1

PIL5 pp CYP707A2

PIL5 pp DFL1

PIL5 pp DWF4

PIL5 pp GA2OX2

PIL5 pp GA3OX1

PIL5 pp GA3OX2

PIL5 pp NCED6

PIL5 pp NCED9

WRKY17 pp LOX2

EIL1 pp EDS16

PIF4 pp TT4

MYB2 pp ADH1

MYB12 pp FLS1

MYB12 pp TT4

ATHB-1 pp ATACO1

MYB26 pp IRX1

MYB26 pp IRX3

ANAC055 pp LOX2

RAP2.3 pp GSTF8

HYH pp TT4

VRN1 pp FT1

EIN3 pp ATACO1

EIN3 pp ATACO2

EIN3 pp EDS16

FUS3 pp GA3OX1

FUS3 pp GA3OX2

MYB30 pp EDS16

OBP1 pp GSTF8

STY1 pp BRU6

STY1 pp DFL1

STY1 pp GH3.3

STY1 pp GH3.4

STY1 pp WES1

PIL6 pp ACS4

PIL6 pp ACS8

DAG1 pp GA3OX1

ATHB-12 pp GA20OX1

KNAT1 pp GA20OX1

AG pp GA3OX1

CBF2 pp ADH1

CBF1 pp GA2OX3

CBF1 pp GA2OX6

CBF1 pp GA3OX1

WRKY11 pp LOX2

MYB32 pp LDOX

SPT pp GA3OX1

SPT pp GA3OX2

MYB4 pp TT4

WRKY62 pp LOX2

MYB29 pp CYP79F1

MYB29 pp CYP79F2

HY5 pp LDOX

HY5 pp TT4

AGL15 pp GA2OX6

ARF7 pp BRU6

ARF7 pp WES1

MYB37 pp DHAR1

TT2 pp LDOX

ARF8 pp DFL1

ARF8 pp GH3.17

GL3 pp FLS1

GL3 pp LDOX

GL3 pp TT4

MYB34 pp AT5G05730

MYB34 pp SUR1

MYB28 pp SUR1

ARF2 pp ACS2

ARF2 pp ACS6

ARF2 pp ACS8

Step 5: Network analysis and community analyses were used to investigate the characteristics of the systemic structure of the sub-interaction network.

NetworkAnalyzer is a Java plugin for Cytoscape, which computes specific parameters that describe the network topology [20]. We used NetworkAnalyzer to determine the number of connected pairs of nodes to examine the overall structure of the GECN model.

Centrality analysis was used for the structural controllability depends on the entire pattern of connections of the AEEPBCN model (Chin et al.,2014). All enzymes have been sorted according to 12 different centrality parameters in order to check which one appears as highly connected nodes using default parameters. (detail information in supplemental information, section II ). It is highly significant that enzymes ( EC:1.14.13.-, EC:1.3.1.-, EC:1.13.11.12, EC:1.14.99.- , EC:1.14.17.4, EC:1.14.11.-, EC:2.3.1.16, EC:2.6.1.57, EC:4.1.1.50, EC:4.1.3.27, EC:4.4.1.14, EC:6.3.-.-) are checked the consistency of existing physiological and genetic studies (Supplementary Information, Additional files 1,section III).

We used the graph clustering algorithm ClusterONE (clustering with overlapping neighborhood expansion), which is available as a plugin to Cytoscape [22], for the association analysis on the EAPCN model. As an unweighted network, ClusterONE automatically tests the value of the transitivity and sets the density threshold to 0.35.
