# Supplementary material for: The Arabidopsis phytohormone crosstalk network involves a consecutive metabolic route and circular control units of transcription factors that regulate enzyme-encoding genes
Source: BMC Syst Biol. 2016 Sep 2;10(1):87. doi: 10.1186/s12918-016-0333-9 (PMC5009710; doi:10.1186/s12918-016-0333-9)
Supplement: Additional file 2: — XML, XGMML, GML, SIF and NNF format of the identified models in the manuscript. (ZIP 164 kb) [file 12918_2016_333_MOESM2_ESM.zip › Additional files 2-SBML format.docx]

**Supplementary file 1_a**: Structural connectivity of the enzyme-based Arabidopsis phytohormones crosstalk network (EAPCN) at the biosynthesis level (xml format).

**Supplementary file 1_b:** Structural connectivity of the enzyme-based Arabidopsis phytohormones crosstalk network (EAPCN) at the biosynthesis level (xgmml format).

**Supplementary file 2_****abscisic_acid_a**: Detailed information of abscisic acid and the crosstalk with other phytohormones in the EAPCN model (xml format).

**Supplementary file 2_abscisic_acid_b**: Detailed information of abscisic acid and the crosstalk with other phytohormones in the EAPCN model (xgmml format).

**Supplementary file 2_****auxin_a**: Detailed information of auxin and the crosstalk with other phytohormones in the EAPCN model (xml format)..

**Supplementary file 2_auxin_b**: Detailed information of auxin and the crosstalk with other phytohormones in the EAPCN model (xgmml format).

**Supplementary file 2_brassinosteroid_a**: Detailed information of brassinosteroid and the crosstalk with other phytohormones in the EAPCN model (xml format)..

**Supplementary file 2_brassinosteroid_b**: Detailed information of brassinosteroid and the crosstalk with other phytohormones in the EAPCN model (xgmml format).

**Supplementary file 2_cytokinin_a**: Detailed information of cytokinin and the crosstalk with other phytohormones in the EAPCN model (xml format)..

**Supplementary file 2_cytokinin_b**: Detailed information of cytokinin and the crosstalk with other phytohormones in the EAPCN model (xgmml format).

**Supplementary file 2_ethylene_a**: Detailed information of ethylene and the crosstalk with other phytohormones in the EAPCN model (xml format).

**Supplementary file 2_ethylene_b**: Detailed information of ethylene and the crosstalk with other phytohormones in the EAPCN model (xgmml format).

**Supplementary file 2_gibberellin_a**: Detailed information of gibberellin and the crosstalk with other phytohormones in the EAPCN model (xml format)..

**Supplementary file 2_gibberellin_b**: Detailed information of gibberellin and the crosstalk with other phytohormones in the EAPCN model (xgmml format).

**Supplementary file 2_jasmonic acid_a**: Detailed information of jasmonic acid and the crosstalk with other phytohormones in the EAPCN model (xml format)..

**Supplementary file 2_jasmonic acid_b:** Detailed information of jasmonic acid and the crosstalk with other phytohormones in the EAPCN model (xgmml format).

**Supplementary file 2_salicylic acid_a**: Detailed information of salicylic acid and the crosstalk with other phytohormones in the EAPCN model (xml format)..

**Supplementary file 2_salicylic acid_b**: Detailed information of salicylic acid and the crosstalk with other phytohormones in the EAPCN model (xgmml format).

**Supplementary file 3_TFs_interactome_network_a:** the transcription factors (TFs)–interactome network(xgmml format).

**Supplementary file 3_TFs_interactome_network_b:** the transcription factors (TFs)–interactome network(gml format).

**Supplementary file 3_TFs_interactome_network_c:** the transcription factors (TFs)–interactome network(sif format).

**Supplementary file 3_TFs_interactome_network_d:** the transcription factors (TFs)–interactome network(nnf format).
