# Supplementary material for: The Arabidopsis phytohormone crosstalk network involves a consecutive metabolic route and circular control units of transcription factors that regulate enzyme-encoding genes
Source: BMC Syst Biol. 2016 Sep 2;10(1):87. doi: 10.1186/s12918-016-0333-9 (PMC5009710; doi:10.1186/s12918-016-0333-9)
Supplement: Additional file 3: — Supplementary Information, section I. Existing physiological and genetic studies. Supplementary Information, section II. Transcription factors-based circular control units of endogenous phytohormone-biosynthetic enzymatic genes in EAPCN. Supplementary Information, section III. Distinct TFs mediating multiple target enzymatic gene of various phytohormone biosynthesis. (DOCX 516 kb) [file 12918_2016_333_MOESM3_ESM.docx]

**Supplementary Information, section I: existing physiological and genetic studies**

**1.** **Crosstalk network of auxin and its crosstalk with other phytohormones**


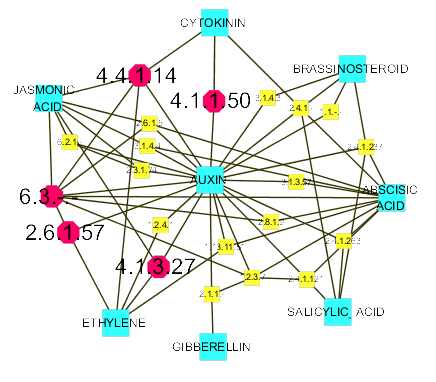


**Figure S1.** Crosstalk network of auxin and its crosstalk with other phytohormones

**Enzyme:** EC: 2.6.1.57 aromatic-amino-acid transaminase

**Crosstalk:** AUXIN –ETHYLENE

**Phytohormone-biosynthetic enzyme gene:** AT1G70560 ( TAA1, WEI8)

**Reference 1 :** PMID: 18394997

Biochemical studies revealed that WEI8 encodes a long-anticipated tryptophan aminotransferase, TAA1, in the essential, yet genetically uncharacterized, indole-3-pyruvic acid (IPA) branch of the auxin biosynthetic pathway. Analysis of TAA1 and its paralogues revealed a link between local auxin production, tissue-specific ethylene effects, and organ development.

**Reference 2 :** PMID: 17630275

The study initially observed that ethylene positively controls auxin biosynthesis in the root apex.Detailed growth studies revealed that the ability of the ethylene precursor 1-aminocyclopropane-1-carboxylic acid to inhibit root cell elongation was significantly enhanced in the presence of auxin. The study conclude that by upregulating auxin biosynthesis, ethylene facilitates its ability to inhibit root cell expansion.

**Reference 3 :** PMID: 17630276

Quantification of the morphological effects of ethylene and auxin in a variety of mutant backgrounds indicates that auxin biosynthesis, transport, signaling, and response are required for the ethylene-induced growth inhibition in roots but not in hypocotyls of dark-grown seedlings. Analysis of the activation of early auxin and ethylene responses at the cellular level, as well as of global changes in gene expression in the wild type versus auxin and ethylene mutants, suggests a simple mechanistic model for the interaction between these two hormones in roots, according to which ethylene and auxin can reciprocally regulate each other's biosyntheses, influence each other's response pathways, and/or act independently on the same target genes. This model not only implies existence of several levels of interaction but also provides a likely explanation for the strong ethylene response defects observed in auxin mutants.

**Enzyme:** EC: 6.3.-.- GH3 enzymes

**Crosstalk :** AUXIN –JASMONIC ACID

**Phytohormone-biosynthetic enzyme gene:** AT2G23170 (GH3.3)

**Reference 1 :** PMID：22730403

The study demonstrate that three auxin-inducible Gretchen Hagen3 (GH3) genes, GH3.3, GH3.5, and GH3.6, act by modulating jasmonic acid homeostasis. The study propose a model in which adventitious rooting is an adaptive developmental response involving crosstalk between the auxin and jasmonate regulatory pathways.

**Enzyme:** EC: 4.1.1.50 adenosylmethionine decarboxylase

**Crosstalk :** AUXIN –CYTOKININ

**Phytohormone-biosynthetic enzyme gene:** AT5G18930

**Reference 1 :** PubMed ID: 20386573

The study demonstrate that BUD2 could be induced by auxin, and the induction is dependent on auxin signaling. The mutation of BUD2 results in hyposensitivity to auxin and hypersensitivity to cytokinin, which is confirmed by callus induction assays. The study suggests that polyamines may play their roles in regulating the plant architecture through affecting the homeostasis of cytokinins and sensitivities to auxin and cytokinin.

**Enzyme:**EC: 4.1.3.27 anthranilate synthase

**Crosstalk :** AUXIN –ETHYLENE- JASMONIC ACID

**Phytohormone-biosynthetic enzyme gene:** AT5G05730 ASA1

**Reference 1 :** PubMed ID: 25524530

The study report that the highly JA-responsive ethylene response factor 109 (ERF109) mediates cross-talk between JA signalling and auxin biosynthesis to regulate lateral root formation in Arabidopsis.ERF109 binds directly to GCC-boxes in the promoters of ASA1 and YUC2, which encode two key enzymes in auxin biosynthesis. Thus, The study reveals a molecular mechanism for JA and auxin cross-talk during JA-induced lateral root formation.

**Reference 2 :** PubMed ID: 19435934

The study further provide evidence showing that the action mechanism of jasmonate to regulate LR formation through ASA1 differs from that of ethylene. The study highlight the importance of ASA1 in jasmonate-induced auxin biosynthesis and reveal a role for jasmonate in the attenuation of auxin transport in the root and the fine-tuning of local auxin distribution in the root basal meristem.

**Enzyme:** EC: 4.4.1.14 1-aminocyclopropane-1-carboxylate synthase

**Crosstalk :** AUXIN –CYTOKININ-ETHYLENE-JASMONIC ACID

**Phytohormone-biosynthetic enzyme gene:** AT1G01480 ACS2, AT-ACC2

**Reference 1 :** PMID:24837830

According to general model, jasmonic acid (JA) and ethylene (ET) signaling pathways are induced in Arabidopsis after an attack of necrotroph, However, abscisic acid (ABA) and salicylic acid (SA) also seem to play a role. In this study, multiple phytohormones quantification and expression analysis of marker genes of the signaling pathways was used to gain a complete view of the interaction of B. napus with S. sclerotiorum. Strong response of ET biosynthetic gene ACS2 was observed, accompanied by increases of SA and JA levels that correspond to the elevated expression of marker genes PR1 and LOX3. Interestingly, the level of ABA and the expression of its marker gene RD26 were also elevated. The results bring the evidence of SA involvement in the interaction of plant with the necrotroph that conflict with the current model.

**Inferred enzymes involved in synergistic as well as antagonistic crosstalks in eight major Arabidopsis phytohormones**

**Enzyme Crosstalk** (1-abscisic acid, 2-auxin, 3-brassinosteroid, 4-cytokinin, 5-ethylene, 6-gibberellin, 7-jasmonic acid 8-salicylic acid)

EC:1.2.4.1 25

EC:3.1.3.57 12

EC: 3.1.4.3 23

EC: 1.1.-.- 23

EC: 2.8.1.9 12

EC: 1.2.3.7 12

EC: 2.1.1.- 26

EC: 6.2.1.- 27

EC: 2.3.1.74 27

EC: 2.6.1.5 127

EC: 1.13.11.51 125

EC: 3.1.4.4 1 27

EC:2.4.1.237 1238

EC:2.4.1.121 128

EC:2.4.1.- 12348

EC: 2.4.1.263 128

**2. Crosstalk network of abscisic acid and its crosstalk with other phytohormones**


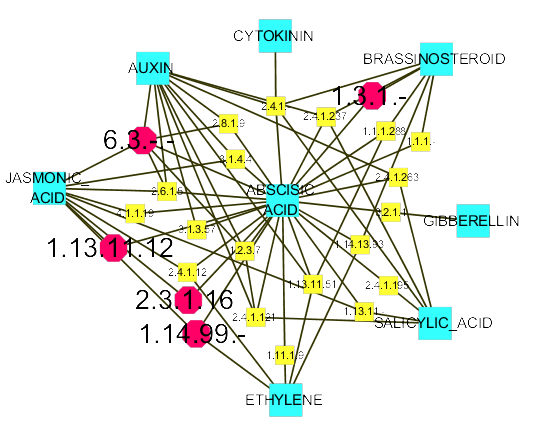


**Figure S2.** Crosstalk network of abscisic acid and its crosstalk with other phytohormones

**Enzyme:EC:** 1.3.1.- With NAD(+) or NADP(+) as acceptor

**Crosstalk :** ABSCISIC ACID - BRASSINOSTEROID

**Phytohormone-biosynthetic enzyme gene:** AT2G38050 ATDET2, DWARF 6, DWF6

**Reference 1 :** PMID: 19526717

The experiments showed that the BR-deficient and BR-insensitive Arabidopsis mutants det2, bri1-5 and bri1-9 were more sensitive to ABA than the wild type (Ws-2), especially the det2 and bri1-9 mutants. Germination, hypocotyl and root elongation, and stomatal apertures of the mutants were more severely inhibited by ABA. All the results suggest that BRs counteract ABA in regulating plant growth, and the interaction may be complicated.

**Enzyme:** EC: 1.13.11.12 linoleate 13S-lipoxygenase

**Crosstalk :** ABSCISIC ACID - JASMONIC ACID

**Phytohormone-biosynthetic enzyme gene:** AT1G17420 ATLOX3, LIPOXYGENASE 3, LOX3

**Reference 1 :**  PMID: 24837830

In this study, multiple phytohormones quantification and expression analysis of marker genes of the signaling pathways was used to gain a complete view of the interaction of B. napus with S. sclerotiorum. accompanied by increases of SA and JA levels that correspond to the elevated expression of marker genes PR1 and LOX3. Interestingly, the level of ABA and the expression of its marker gene RD26 were also elevated. Furthermore, induction of the SA-dependent defense decreased disease symptoms. In addition, SA signaling is suggested as a possible target for manipulation by S. sclerotiorum.

**Phytohormone-biosynthetic enzyme gene:** AT1G67560 ATLOX6, LIPOXYGENASE 6, LOX6

**Reference 1 :** PMID: 25789569

hormone profiling together with gene expression of key enzymes involved in abscisic acid (ABA) and jasmonate biosynthesis were studied in desiccating Arabidopsis roots. Jasmonic acid (JA) content transiently increased after stress imposition whereas progressive and concomitant ABA and Jasmonoyl Isoleucine (JA-Ile) accumulations were detected. Molecular data suggest that, at least, part of the hormonal regulation takes place at the biosynthetic level. These observations also point to a possible involvement of jasmonates on ABA biosynthesis under stress. To test this hypothesis, mutants impaired in jasmonate biosynthesis (opr3, lox6 and jar1-1) and in JA-dependent signalling (coi1) were employed. Results showed that the early JA accumulation leading to JA-Ile build up was necessary for an ABA increase in roots under two different water stress conditions. Signal transduction between water stress-induced JA-Ile accumulation and COI1 is necessary for a full induction of the ABA biosynthesis pathway and subsequent hormone accumulation in roots of Arabidopsis plants. The present work adds a level of interaction between jasmonates and ABA at the biosynthetic level.

**Enzyme:EC: 2.3.1.16** acetyl-CoA C-acyltransferase

**Crosstalk :** ABSCISIC ACID - JASMONIC ACID

**Phytohormone-biosynthetic enzyme gene:** AT2G33150 KAT2, PED1, PEROXISOMAL 3-KETOACYL-COA THIOLASE 3, PEROXISOME DEFECTIVE 1, PKT3

**Reference 1 :** PMID: 21257607

The study report that an enzyme catalyzing β-oxidation of fatty acids, 3-ketoacyl-CoA thiolase-2 (KAT2/PED1/PKT3) (EC 2.3.1.16), is involved in ABA signaling. The study provide genetic evidence that KAT2 positively regulates ABA signaling in all the major ABA responses, including ABA-induced inhibition of seed germination and post-germination growth arrest, and ABA-induced stomatal closure and stomatal opening inhibition in Arabidopsis thaliana. KAT2 was shown to be important for reactive oxygen species (ROS) production in response to ABA, suggesting that KAT2 regulates ABA signaling at least partly through modulating ROS homeostasis in plant cells. Additionally, The study provide data suggesting that KAT2 may function downstream of an important WRKY transcription repressor WRKY40, which may link KAT2 with the ABA receptor ABAR/CHLH-mediated signaling.

**Enzyme:EC: 1.14.99.-** Miscellaneous (requires further characterization)

**Crosstalk :** ABSCISIC ACID-ETHYLENE- JASMONIC ACID

**Phytohormone-biosynthetic enzyme gene:** AT3G63520 ATNCED1, CAROTENOID CLEAVAGE DIOXYGENASE 1, CCD1, NCED1

**Reference 1** : PMID: 16317577

The study have confirmed that at high concentrations, 2,4-D induced the expression of the gene NCED1, which encodes 9-cis-epoxycarotenoid dioxygenase, a key regulatory enzyme of ABA biosynthesis. To understand the concentration-dependent mode of action of 2,4-D, The study further examined the regulation of whole genome of Arabidopsis in response to a range of 2,4-D concentrations from 0.001 to 1.0 mM, using the ATH1-121501 Arabidopsis whole genome microarray developed by Affymetrix. Results of this study indicated that 2,4-D induced the expression of auxin-response genes (IAA1, IAA13, IAA19) at both auxinic and herbicidal levels of application, whereas the TIR1 and ASK1 genes, which are associated with ubiquitin-mediated auxin signalling, were down-regulated in response to low concentrations of 2,4-D application. It was also observed that in response to low concentrations of 2,4-D, ethylene biosynthesis was induced, as suggested by the up-regulation of genes encoding 1-aminocyclopropane-1-carboxylic acid (ACC) synthase and ACC oxidase. Although genes involved in ethylene biosynthesis were not regulated in response to 0.1 and 1.0 mM 2,4-D, ethylene signalling was induced as indicated by the down-regulation of CTR1 and ERS, both of which play a key role in the ethylene signalling pathway. In response to 1.0 mM 2,4-D, both ABA biosynthesis and signalling were induced, in contrast to the response to lower concentrations of 2,4-D where ABA biosynthesis was suppressed. The study present a comprehensive model indicating a molecular mode of action for 2,4-D in Arabidopsis and the effects of this growth regulator on the auxin, ethylene and abscisic acid pathways.

**Enzyme:** EC:6.3.-.- GH3 enzymes

**Crosstalk :** ABSCISIC ACID--AUXIN- JASMONIC ACID

**Phytohormone-biosynthetic enzyme gene:** AT4G27260.1 (GH3.5)

**Reference 1 :** PubMed ID：19704630

The study have demonstrated that auxin homeostasis directly links growth regulation with stress adaptation responses through interactions with salicylic acid (SA) and abscisic acid (ABA) signals. In this signaling network, the endogenous auxin content is coordinately regulated through negative feedback by a group of auxin-inducible GH3 genes that encode auxin-conjugating enzymes.

**Inferred enzymes involved in synergistic as well as antagonistic crosstalks in eight major Arabidopsis phytohormones**

**Enzyme Crosstalk** (1-abscisic acid, 2-auxin, 3-brassinosteroid, 4-cytokinin, 5-ethylene, 6-gibberellin, 7-jasmonic acid 8-salicylic acid)

EC: 1.11.1.9 15

EC: 1.1.1.- 13

EC: 2.4.1.195 18

EC: 4.1.1.19 17

EC: 1.1.1.288 13

EC: 3.2.1.1 16

EC: 3.1.3.57 12

EC: 1.2.3.7 12

EC: 2.8.1.9 12

EC: 2.4.1.121 128

EC: 2.4.1.237 1238

EC: 3.1.4.4 127

EC: 2.4.1.263 128

EC: 2.4.1.- 12348

EC: 1.13.11.- 178

EC: 1.14.13.93 135

EC: 2.4.1.12 157

EC: 1.13.11.51 125

EC: 2.6.1.5 127

**3. Crosstalk network of brassinosteroid and its crosstalk with other phytohormones**


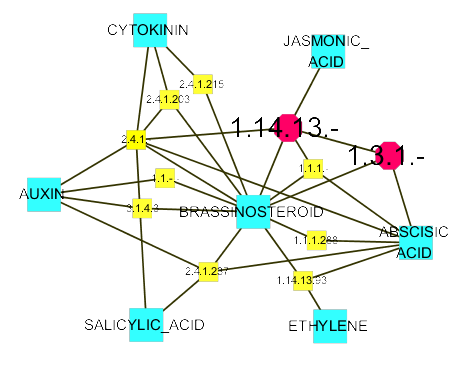


**Figure S3.** Crosstalk network of brassinosteroid and its crosstalk with other phytohormones

**Enzyme:** EC: 1.14.13.- With NADH or NADPH as one donor, and incorporation of one atom of oxygen

**Crosstalk :** BRASSINOSTEROID- JASMONIC ACID

**Phytohormone-biosynthetic enzyme gene:** AT3G50660 DWF4,

**Reference 1**: PMID: 23297052

The study propose a model in which BR and JA cooperate to balance energy allocation between growth and defense responses. In ambient conditions, BRs promote plant growth; however, when stresses trigger JA signaling, JA compromises BR signaling by downregulating DWF4 expression.

**Reference 2 :** PubMed ID 19741050

The results indicate that (i) BR is involved in JA signaling and negatively regulates JA inhibition of root growth, and (ii) the DWF4 is down-regulated by JA and is located downstream of COI1 in the JA signaling pathway.

**Enzyme:** EC: 1.3.1.- With NAD(+) or NADP(+) as acceptor

**Crosstalk :** ABSCISIC ACID - BRASSINOSTEROID

**Phytohormone-biosynthetic enzyme gene:** AT2G38050 ATDET2, DWARF 6, DWF6

**Reference 1 :** PMID: 19526717

The experiments showed that the BR-deficient and BR-insensitive Arabidopsis mutants det2, bri1-5 and bri1-9 were more sensitive to ABA than the wild type (Ws-2), especially the det2 and bri1-9 mutants. Germination, hypocotyl and root elongation, and stomatal apertures of the mutants were more severely inhibited by ABA. All the results suggest that BRs counteract ABA in regulating plant growth, and the interaction may be complicated.

**Inferred enzymes involved in synergistic as well as antagonistic crosstalks in eight major Arabidopsis phytohormones**

**Enzyme Crosstalk** (1-abscisic acid, 2-auxin, 3-brassinosteroid, 4-cytokinin, 5-ethylene, 6-gibberellin, 7-jasmonic acid 8-salicylic acid)

EC: 3.1.4.3 23

EC: 2.4.1.215 34

EC: 1.1.1.- 13

EC: 1.1.1.288 13

EC: 1.1.-.- 23

EC: 2.4.1.203 34

EC: 1.14.13.93 135

EC: 1.13.11.51 125

EC: 2.4.1.237 1238

EC: 2.4.1.- 12348

**4. Crosstalk network of cytokinin and its crosstalk with other phytohormones**


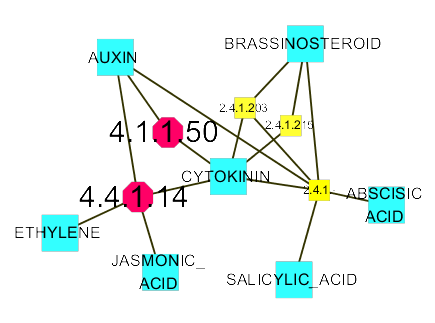


**Figure S4.** Crosstalk network of cytokinin and its crosstalk with other phytohormones

**Enzyme:** EC: 4.1.1.50 adenosylmethionine decarboxylase

**Crosstalk :** AUXIN –CYTOKININ

**Phytohormone-biosynthetic enzyme gene:** AT5G18930

**Reference 1 :** PubMed ID: 20386573

The study demonstrate that BUD2 could be induced by auxin, and the induction is dependent on auxin signaling. The mutation of BUD2 results in hyposensitivity to auxin and hypersensitivity to cytokinin, which is confirmed by callus induction assays. The study suggests that polyamines may play their roles in regulating the plant architecture through affecting the homeostasis of cytokinins and sensitivities to auxin and cytokinin.

**Enzyme:** EC: 4.4.1.14 1-aminocyclopropane-1-carboxylate synthase

**Crosstalk :** AUXIN –CYTOKININ-ETHYLENE- JASMONIC ACID

**Phytohormone-biosynthetic enzyme gene:** AT1G01480 ACS2, AT-ACC2

**Reference 1 :** PMID:24837830

According to general model, jasmonic acid (JA) and ethylene (ET) signaling pathways are induced in Arabidopsis after an attack of necrotroph, However, abscisic acid (ABA) and salicylic acid (SA) also seem to play a role. In this study, multiple phytohormones quantification and expression analysis of marker genes of the signaling pathways was used to gain a complete view of the interaction of B. napus with S. sclerotiorum. Strong response of ET biosynthetic gene ACS2 was observed, accompanied by increases of SA and JA levels that correspond to the elevated expression of marker genes PR1 and LOX3. Interestingly, the level of ABA and the expression of its marker gene RD26 were also elevated. The results bring the evidence of SA involvement in the interaction of plant with the necrotroph that conflict with the current model.

**Inferred enzymes involved in synergistic as well as antagonistic crosstalks in eight major Arabidopsis phytohormones**

**Enzyme Crosstalk** (1-abscisic acid, 2-auxin, 3-brassinosteroid, 4-cytokinin, 5-ethylene, 6-gibberellin, 7-jasmonic acid 8-salicylic acid)

EC: 2.4.1.215 34

EC: 2.4.1.203 34

EC: 2.4.1.- 12348

**5. Crosstalk network of ethylene and its crosstalk with other phytohormones**


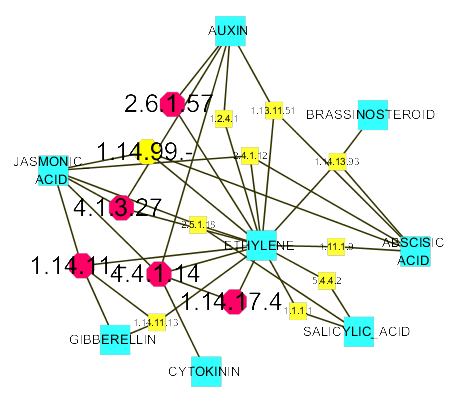


**Figure S5.** Crosstalk network of ethylene and its crosstalk with other phytohormones

**Enzyme:** EC: 2.6.1.57 aromatic-amino-acid transaminase

**Crosstalk :** AUXIN –ETHYLENE

**Phytohormone-biosynthetic enzyme gene:**  AT1G70560 ( TAA1, WEI8)

**Reference 1 :** PMID: 18394997

Biochemical studies revealed that WEI8 encodes a long-anticipated tryptophan aminotransferase, TAA1, in the essential, yet genetically uncharacterized, indole-3-pyruvic acid (IPA) branch of the auxin biosynthetic pathway. Analysis of TAA1 and its paralogues revealed a link between local auxin production, tissue-specific ethylene effects, and organ development.

**Reference 2** : PMID: 17630275

The study initially observed that ethylene positively controls auxin biosynthesis in the root apex. The study subsequently demonstrated that ethylene-regulated root growth is dependent on (1) the transport of auxin from the root apex via the lateral root cap and (2) auxin responses occurring in multiple elongation zone tissues. Detailed growth studies revealed that the ability of the ethylene precursor 1-aminocyclopropane-1-carboxylic acid to inhibit root cell elongation was significantly enhanced in the presence of auxin. The study conclude that by upregulating auxin biosynthesis, ethylene facilitates its ability to inhibit root cell expansion.

**Reference 3 :** PMID: 17630276

Quantification of the morphological effects of ethylene and auxin in a variety of mutant backgrounds indicates that auxin biosynthesis, transport, signaling, and response are required for the ethylene-induced growth inhibition in roots but not in hypocotyls of dark-grown seedlings. Analysis of the activation of early auxin and ethylene responses at the cellular level, as well as of global changes in gene expression in the wild type versus auxin and ethylene mutants, suggests a simple mechanistic model for the interaction between these two hormones in roots, according to which ethylene and auxin can reciprocally regulate each other's biosyntheses, influence each other's response pathways, and/or act independently on the same target genes. This model not only implies existence of several levels of interaction but also provides a likely explanation for the strong ethylene response defects observed in auxin mutants.

**Enzyme:** EC: 1.14.17.4 aminocyclopropanecarboxylate oxidase

**Crosstalk :** ETHYLENE - ***

**Phytohormone-biosynthetic enzyme gene:** AT1G62380

**Reference 1 :** PubMed ID : 20023197

The study show that ethylene promotes endosperm cap weakening of Lepidium and endosperm rupture of both species and that it counteracts the inhibitory action of abscisic acid (ABA) on these two processes. Cross-species microarrays of the Lepidium micropylar endosperm cap and the radicle show that the ethylene-ABA antagonism involves both tissues and has the micropylar endosperm cap as a major target. Ethylene counteracts the ABA-induced inhibition without affecting seed ABA levels. The Arabidopsis loss-of-function mutants ACC oxidase2 (aco2; ethylene biosynthesis) and constitutive triple response1 (ethylene signaling) are impaired in the 1-aminocyclopropane-1-carboxylic acid (ACC)-mediated reversion of the ABA-induced inhibition of seed germination. Ethylene production by the ACC oxidase orthologs Lepidium ACO2 and Arabidopsis ACO2 appears to be a key regulatory step. Endosperm cap weakening and rupture are promoted by ethylene and inhibited by ABA to regulate germination in a process conserved across the Brassicaceae.

**Phytohormone-biosynthetic enzyme gene:**  AT1G12010

**Reference 1 :** PMID: 19513806

These data further suggest that ABA and ethylene may control the hormonal biosynthesis, catabolism, or signaling of each other to enhance their antagonistic effects upon seed germination and early seedling growth.

**Enzyme:** EC: 4.4.1.14 1-aminocyclopropane-1-carboxylate synthase

**Crosstalk :** AUXIN –CYTOKININ-ETHYLENE- JASMONIC ACID

**Phytohormone-biosynthetic enzyme gene:** AT1G01480 ACS2, AT-ACC2

**Reference 1 :** PMID:24837830

According to general model, jasmonic acid (JA) and ethylene (ET) signaling pathways are induced in Arabidopsis after an attack of necrotroph, However, abscisic acid (ABA) and salicylic acid (SA) also seem to play a role. In this study, multiple phytohormones quantification and expression analysis of marker genes of the signaling pathways was used to gain a complete view of the interaction of B. napus with S. sclerotiorum. Strong response of ET biosynthetic gene ACS2 was observed, accompanied by increases of SA and JA levels that correspond to the elevated expression of marker genes PR1 and LOX3. Interestingly, the level of ABA and the expression of its marker gene RD26 were also elevated. The results bring the evidence of SA involvement in the interaction of plant with the necrotroph that conflict with the current model.

**Enzyme:** EC: 4.1.3.27 anthranilate synthase

**Crosstalk :** AUXIN –ETHYLENE- JASMONIC ACID

**Phytohormone-biosynthetic enzyme gene:** AT5G05730 ASA1

**Reference 1 :** PubMed ID: 25524530

The study report that the highly JA-responsive ethylene response factor 109 (ERF109) mediates cross-talk between JA signalling and auxin biosynthesis to regulate lateral root formation in Arabidopsis.ERF109 binds directly to GCC-boxes in the promoters of ASA1 and YUC2, which encode two key enzymes in auxin biosynthesis. Thus, The study reveals a molecular mechanism for JA and auxin cross-talk during JA-induced lateral root formation.

**Reference 2 :** PubMed ID: 19435934

The study further provide evidence showing that the action mechanism of jasmonate to regulate LR formation through ASA1 differs from that of ethylene. The study highlight the importance of ASA1 in jasmonate-induced auxin biosynthesis and reveal a role for jasmonate in the attenuation of auxin transport in the root and the fine-tuning of local auxin distribution in the root basal meristem.

**Inferred enzymes involved in synergistic as well as antagonistic crosstalks in eight major Arabidopsis phytohormones**

**Enzyme Crosstalk** (1-abscisic acid, 2-auxin, 3-brassinosteroid, 4-cytokinin, 5-ethylene, 6-gibberellin, 7-jasmonic acid 8-salicylic acid)

EC: 1.14.11.- 567

EC: 1.11.1.9 15

EC: 5.4.4.2 58

EC: 1.2.4.1 25

EC: 1.14.11.13 56

EC: 1.1.1.1 58

EC: 2.5.1.18 578

EC: 2.4.1.12 157

EC: 1.14.13.93 135

EC: 1.13.11.51 125

**6. Crosstalk network of gibberellin and its crosstalk with other phytohormones**


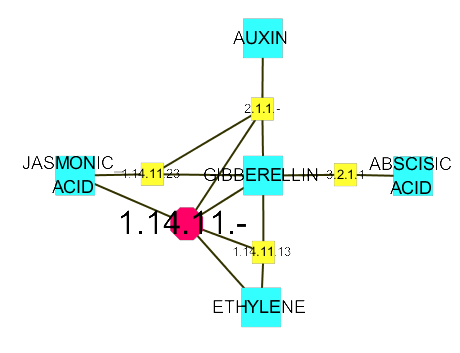


**Figure S6.** Crosstalk network of gibberellin and its crosstalk with other phytohormones

**Enzyme:** EC: 1.14.11.- With 2-oxoglutarate as one donor, and incorporation of one atom each of oxygen into both donors

**Crosstalk :** ETHYLENE-GIBBERELLIN-JASMONIC ACID

**Phytohormone-biosynthetic enzyme gene:** AT1G78440

**Reference 1 :** PubMed ID 19325888

The study conclude that GA promotes JA biosynthesis to control the expression of MYB21, MYB24, and MYB57. Therefore, we have established a hierarchical relationship between GA and JA in that modulation of JA pathway by GA is one of the prerequisites for GA to regulate the normal stamen development in Arabidopsis.

**Phytohormone-biosynthetic enzyme gene:** AT4G22880

**Reference 1 :** PMID: 21545406

The results suggested that BR affects JA-induced anthocyanin accumulation by regulating the 'late' anthocyanin biosynthesis genes and this regulation might be mediated by the WD-repeat/Myb/bHLH transcriptional complexes.

**Inferred enzymes involved in synergistic as well as antagonistic crosstalks in eight major Arabidopsis phytohormones**

**Enzyme Crosstalk** (1-abscisic acid, 2-auxin, 3-brassinosteroid, 4-cytokinin, 5-ethylene, 6-gibberellin, 7-jasmonic acid 8-salicylic acid)

EC: 1.14.11.13 56

EC: 2.1.1.- 26

EC: 3.2.1.1 16

EC: 1.14.11.23 67

**7. Crosstalk network of jasmonic acid and its crosstalk with other phytohormones**


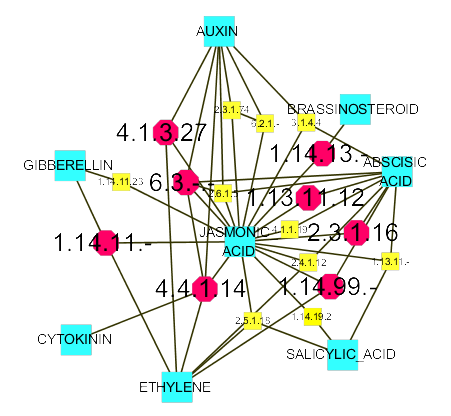


**Figure S7.** Crosstalk network of jasmonic acid and its crosstalk with other phytohormones

**Enzyme:** EC: 1.13.11.12 linoleate 13S-lipoxygenase

**Crosstalk :** ABSCISIC ACID - JASMONIC ACID

**Phytohormone-biosynthetic enzyme gene:** AT1G17420 ATLOX3, LIPOXYGENASE 3, LOX3

**Reference 1 :**  PMID: 24837830

In this study, multiple phytohormones quantification and expression analysis of marker genes of the signaling pathways was used to gain a complete view of the interaction of B. napus with S. sclerotiorum. accompanied by increases of SA and JA levels that correspond to the elevated expression of marker genes PR1 and LOX3. Interestingly, the level of ABA and the expression of its marker gene RD26 were also elevated. Furthermore, induction of the SA-dependent defense decreased disease symptoms. In addition, SA signaling is suggested as a possible target for manipulation by S. sclerotiorum.

**Gene encoded the enzymes:** AT1G67560　 ATLOX6, LIPOXYGENASE 6, LOX6

**Reference 1 :** PMID: 25789569

hormone profiling together with gene expression of key enzymes involved in abscisic acid (ABA) and jasmonate biosynthesis were studied in desiccating Arabidopsis roots. Jasmonic acid (JA) content transiently increased after stress imposition whereas progressive and concomitant ABA and Jasmonoyl Isoleucine (JA-Ile) accumulations were detected. Molecular data suggest that, at least, part of the hormonal regulation takes place at the biosynthetic level. These observations also point to a possible involvement of jasmonates on ABA biosynthesis under stress. To test this hypothesis, mutants impaired in jasmonate biosynthesis (opr3, lox6 and jar1-1) and in JA-dependent signalling (coi1) were employed. Results showed that the early JA accumulation leading to JA-Ile build up was necessary for an ABA increase in roots under two different water stress conditions. Signal transduction between water stress-induced JA-Ile accumulation and COI1 is necessary for a full induction of the ABA biosynthesis pathway and subsequent hormone accumulation in roots of Arabidopsis plants. The present work adds a level of interaction between jasmonates and ABA at the biosynthetic level.

**Enzyme:EC:** 1.14.13.-

**Crosstalk :** BRASSINOSTEROID- JASMONIC ACID

**Phytohormone-biosynthetic enzyme gene:** AT3G50660 DWF4,

**Reference 1:** PMID: 23297052

The study propose a model in which BR and JA cooperate to balance energy allocation between growth and defense responses. In ambient conditions, BRs promote plant growth; however, when stresses trigger JA signaling, JA compromises BR signaling by downregulating DWF4 expression.

**Reference 2 :** PubMed ID 19741050

The results indicate that (i) BR is involved in JA signaling and negatively regulates JA inhibition of root growth, and (ii) the DWF4 is down-regulated by JA and is located downstream of COI1 in the JA signaling pathway.

**Enzyme:** EC: 2.3.1.16 acetyl-CoA C-acyltransferase

**Crosstalk :** ABSCISIC ACID - JASMONIC ACID

**Phytohormone-biosynthetic enzyme gene:** AT2G33150 KAT2, PED1, PEROXISOMAL 3-KETOACYL-COA THIOLASE 3, PEROXISOME DEFECTIVE 1, PKT3

**Reference 1 :** PMID: 21257607

The study report that an enzyme catalyzing β-oxidation of fatty acids, 3-ketoacyl-CoA thiolase-2 (KAT2/PED1/PKT3) (EC 2.3.1.16), is involved in ABA signaling. The study provide genetic evidence that KAT2 positively regulates ABA signaling in all the major ABA responses, including ABA-induced inhibition of seed germination and post-germination growth arrest, and ABA-induced stomatal closure and stomatal opening inhibition in Arabidopsis thaliana. KAT2 was shown to be important for reactive oxygen species (ROS) production in response to ABA, suggesting that KAT2 regulates ABA signaling at least partly through modulating ROS homeostasis in plant cells. Additionally, The study provide data suggesting that KAT2 may function downstream of an important WRKY transcription repressor WRKY40, which may link KAT2 with the ABA receptor ABAR/CHLH-mediated signaling.

**Enzyme:** EC: 6.3.-.- GH3 enzymes

**Crosstalk :** AUXIN –JASMONIC ACID

**Phytohormone-biosynthetic enzyme gene:** AT2G23170 (GH3.3)

**Reference 1 :** PubMed ID：22730403

The study demonstrate that three auxin-inducible Gretchen Hagen3 (GH3) genes, GH3.3, GH3.5, and GH3.6, act by modulating jasmonic acid homeostasis. The study propose a model in which adventitious rooting is an adaptive developmental response involving crosstalk between the auxin and jasmonate regulatory pathways.

**Enzyme:** EC: 4.1.3.27 anthranilate synthase

**Crosstalk :** AUXIN –ETHYLENE- JASMONIC ACID

**Phytohormone-biosynthetic enzyme gene:** AT5G05730 ASA1

**Reference 1 :** PubMed ID: 25524530

The study report that the highly JA-responsive ethylene response factor 109 (ERF109) mediates cross-talk between JA signalling and auxin biosynthesis to regulate lateral root formation in Arabidopsis.ERF109 binds directly to GCC-boxes in the promoters of ASA1 and YUC2, which encode two key enzymes in auxin biosynthesis. Thus, The study reveals a molecular mechanism for JA and auxin cross-talk during JA-induced lateral root formation.

**Reference 2 :** PubMed ID: 19435934

The study further provide evidence showing that the action mechanism of jasmonate to regulate LR formation through ASA1 differs from that of ethylene. The study highlight the importance of ASA1 in jasmonate-induced auxin biosynthesis and reveal a role for jasmonate in the attenuation of auxin transport in the root and the fine-tuning of local auxin distribution in the root basal meristem.

**Enzyme:** EC: 4.4.1.14 1-aminocyclopropane-1-carboxylate synthase

**Crosstalk :** AUXIN –CYTOKININ-ETHYLENE- JASMONIC ACID

**Phytohormone-biosynthetic enzyme gene:** AT1G01480 ACS2, AT-ACC2

**Reference 1** : PMID:24837830

According to general model, jasmonic acid (JA) and ethylene (ET) signaling pathways are induced in Arabidopsis after an attack of necrotroph, However, abscisic acid (ABA) and salicylic acid (SA) also seem to play a role. In this study, multiple phytohormones quantification and expression analysis of marker genes of the signaling pathways was used to gain a complete view of the interaction of B. napus with S. sclerotiorum. Strong response of ET biosynthetic gene ACS2 was observed, accompanied by increases of SA and JA levels that correspond to the elevated expression of marker genes PR1 and LOX3. Interestingly, the level of ABA and the expression of its marker gene RD26 were also elevated. The results bring the evidence of SA involvement in the interaction of plant with the necrotroph that conflict with the current model.

**Enzyme:** EC: 1.14.11.- With 2-oxoglutarate as one donor, and incorporation of one atom each of oxygen into both donors

**Crosstalk :** ETHYLENE-GIBBERELLIN-JASMONIC ACID

**Phytohormone-biosynthetic enzyme gene:** AT1G78440

**Reference 1** : PubMed ID 19325888

The study conclude that GA promotes JA biosynthesis to control the expression of MYB21, MYB24, and MYB57. Therefore, we have established a hierarchical relationship between GA and JA in that modulation of JA pathway by GA is one of the prerequisites for GA to regulate the normal stamen development in Arabidopsis.

**Gene encoded the enzymes:** AT4G22880

**Reference 1 :** PMID: 21545406

The results suggested that BR affects JA-induced anthocyanin accumulation by regulating the 'late' anthocyanin biosynthesis genes and this regulation might be mediated by the WD-repeat/Myb/bHLH transcriptional complexes.

**Enzyme:** EC: 1.14.99.- Miscellaneous (requires further characterization)

**Crosstalk :** ABSCISIC ACID-ETHYLENE- JASMONIC ACID

**Phytohormone-biosynthetic enzyme gene:** AT3G63520 ATNCED1, CAROTENOID CLEAVAGE DIOXYGENASE 1, CCD1, NCED1

**Reference 1 :** PMID: 16317577

The study have confirmed that at high concentrations, 2,4-D induced the expression of the gene NCED1, which encodes 9-cis-epoxycarotenoid dioxygenase, a key regulatory enzyme of ABA biosynthesis. To understand the concentration-dependent mode of action of 2,4-D, The study further examined the regulation of whole genome of Arabidopsis in response to a range of 2,4-D concentrations from 0.001 to 1.0 mM, using the ATH1-121501 Arabidopsis whole genome microarray developed by Affymetrix. Results of this study indicated that 2,4-D induced the expression of auxin-response genes (IAA1, IAA13, IAA19) at both auxinic and herbicidal levels of application, whereas the TIR1 and ASK1 genes, which are associated with ubiquitin-mediated auxin signalling, were down-regulated in response to low concentrations of 2,4-D application. It was also observed that in response to low concentrations of 2,4-D, ethylene biosynthesis was induced, as suggested by the up-regulation of genes encoding 1-aminocyclopropane-1-carboxylic acid (ACC) synthase and ACC oxidase. Although genes involved in ethylene biosynthesis were not regulated in response to 0.1 and 1.0 mM 2,4-D, ethylene signalling was induced as indicated by the down-regulation of CTR1 and ERS, both of which play a key role in the ethylene signalling pathway. In response to 1.0 mM 2,4-D, both ABA biosynthesis and signalling were induced, in contrast to the response to lower concentrations of 2,4-D where ABA biosynthesis was suppressed. The study present a comprehensive model indicating a molecular mode of action for 2,4-D in Arabidopsis and the effects of this growth regulator on the auxin, ethylene and abscisic acid pathways.

**Inferred enzymes involved in synergistic as well as antagonistic crosstalks in eight major Arabidopsis phytohormones**

**Enzyme Crosstalk** (1-abscisic acid, 2-auxin, 3-brassinosteroid, 4-cytokinin, 5-ethylene, 6-gibberellin, 7-jasmonic acid 8-salicylic acid)

EC: 2.3.1.74 27

EC: 1.14.19.2 78

EC: 6.2.1.- 27

EC:1.14.11.23 67

EC: 4.1.1.19 17

EC: 2.4.1.12 157

EC: 3.1.4.4 127

EC:2.6.1.5 127

EC:2.5.1.18 578

EC:1.13.11.- 178

**8. Crosstalk network of salicylic acid and its crosstalk with other phytohormones**


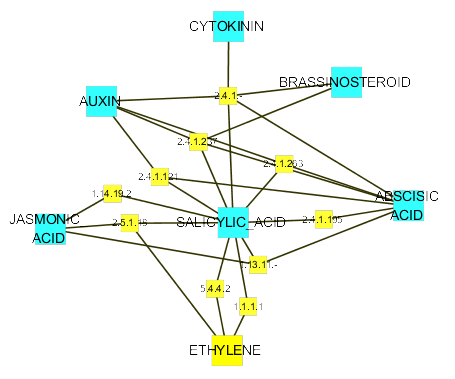


**Figure S8.** Crosstalk network of salicylic acid and its crosstalk with other phytohormones

**Inferred enzymes involved in synergistic as well as antagonistic crosstalks in eight major Arabidopsis phytohormones**

**Enzyme Crosstalk** (1-abscisic acid, 2-auxin, 3-brassinosteroid, 4-cytokinin, 5-ethylene, 6-gibberellin, 7-jasmonic acid 8-salicylic acid)

EC: 5.4.4.2 58

EC:1.14.19.2 78

EC:2.4.1.195 18

EC:1.1.1.1 58

EC:2.4.1.121 128

EC:2.5.1.18 578

EC:2.4.1.237 1238

EC:1.13.11.- 178

EC:2.4.1.263 128

EC: 2.4.1.- 12348

**Supplementary Information, section II: the transcription factors-based circular control units of endogenous phytohormone-biosynthetic enzymatic genes in EAPCN**


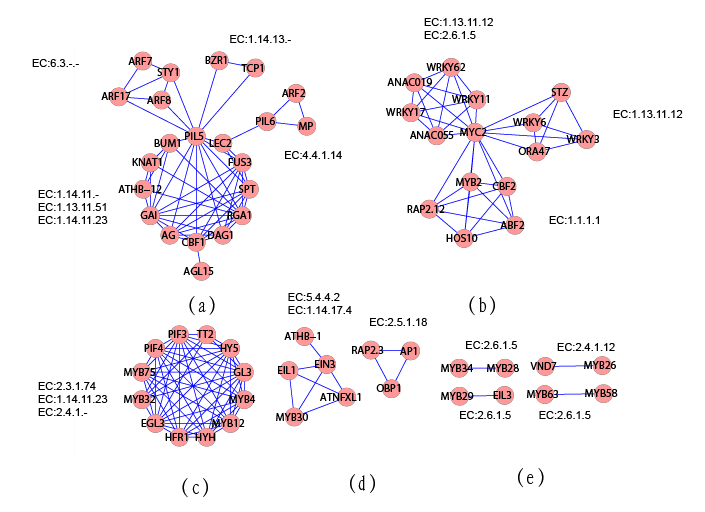


Figure S9: **Subset of TFs regulating enzymatic genes related to phytohormones crosstalk in** EAPCN**. In the network, vertices nodes present the transcription factors (TFs), and edges means two TFs have the same target gene.**

**(**1-abscisic acid, 2-auxin, 3-brassinosteroid, 4-cytokinin, 5-ethylene, 6-gibberellin, 7-jasmonic acid 8-salicylic acid)

**(a): TFs Phytohormone-biosynthetic enzyme gene Enzyme Crosstalk**  SPT(AT4G36930) GA3OX2 (AT1G80340) 1.14.11.- 567

LEC2(AT1G28300) GA3OX2 (AT1G80340) 1.14.11.- 567

FUS3(AT3G26790) GA3OX2 (AT1G80340) 1.14.11.- 567

AG (AT4G18960) GA3OX1(AT1G15550) 1.14.11.- 567

RGA1 (AT2G01570) GA3OX1(AT1G15550) 1.14.11.- 567

DAG1(AT3G61850) GA3OX1(AT1G15550) 1.14.11.- 567

CBF1(AT4G25490) GA3OX1(AT1G15550) 1.14.11.- 567

GAI (AT1G14920) GA3OX1(AT1G15550) 1.14.11.- 567

KNAT1(AT4G08150) GA20OX1(AT4G25420) 1.14.11.- 567

BUM1(AT1G62360) GA2OX4 (AT1G47990) 1.14.11.- 567

ATHB-12(AT3G61890) GA20OX1(AT4G25420) 1.14.11.23 67

PIL5(AT2G20180) NCED9(AT1G78390) 1.13.11.51 125

**TFs Phytohormone-biosynthetic enzyme gene Enzyme Crosstalk**

STY1(AT3G51060) WES1 (AT4G27260)  6.3.-.- 127

ARF7(AT5G20730)  WES1 (AT4G27260) 6.3.-.- 127

ARF17(AT1G77850) WES1 (AT4G27260) 6.3.-.- 127

ARF8(AT5G37020) GH3.17 (AT1G28130) 6.3.-.- 127

**TFs Phytohormone-biosynthetic enzyme gene Enzyme Crosstalk**

BZR1(AT1G75080) DWF4 (AT3G50660) 1.14.13.- 37

TCP1(AT1G67260) DWF4 (AT3G50660) 1.14.13.- 37

**TFs Phytohormone-biosynthetic enzyme gene Enzyme Crosstalk**

MP(AT1G19850) ACS8 (AT4G37770) 4.4.1.14 2457

PIL6(AT3G59060)  ACS8 (AT4G37770) 4.4.1.14 2457

ARF2(AT5G62000) ACS8 (AT4G37770) 4.4.1.14 2457

**(b): TFs Phytohormone-biosynthetic enzyme gene Enzyme Crosstalk**

WRKY17 (AT2G24570) LOX2(AT3G45140) 1.13.11.12 17

WRKY62 (AT5G01900) LOX2(AT3G45140) 1.13.11.12 17

ANAC019(AT1G52890) LOX2(AT3G45140) 1.13.11.12 17

ANAC055(AT3G15500) LOX2(AT3G45140) 1.13.11.12 17

WRKY11(AT4G31550) LOX2(AT3G45140) 1.13.11.12 17

MYC2(AT1G32640) TAT3 (AT2G24850) 2.6.1.5 1 27

**TFs Phytohormone-biosynthetic enzyme gene Enzyme Crosstalk**

MYB2(AT2G47190) ADH1(AT1G77120) 1.1.1.1 58

RAP2.12(AT1G53910) ADH1(AT1G77120) 1.1.1.1 58

ABF2(AT1G45249) ADH1(AT1G77120) 1.1.1.1 58

HOS10(AT1G35515) ADH1(AT1G77120) 1.1.1.1 58

CBF2(AT4G25470) ADH1(AT1G77120) 1.1.1.1 58

**TFs Phytohormone-biosynthetic enzyme gene Enzyme Crosstalk**

WRKY3(AT2G03340) LOX3(AT1G17420) 1.13.11.12 17

WRKY6(AT1G62300) LOX3(AT1G17420) 1.13.11.12 17

STZ(AT1G27730) LOX3(AT1G17420) 1.13.11.12 17

ORA47(AT1G74930) LOX3(AT1G17420) 1.13.11.12 17

**(c) : TFs Phytohormone-biosynthetic enzyme gene Enzyme Crosstalk**

TT2(AT5G35550) LDOX(AT4G22880) 1.14.11.23 67

MYB4(AT4G38620) TT4(AT5G13930) 2.3.1.74 27

HFR1(AT1G02340) TT4(AT5G13930) 2.3.1.74 27

HY5(AT5G11260) TT4(AT5G13930) 2.3.1.74 27

PIF4(AT2G43010) TT4(AT5G13930) 2.3.1.74 27

HYH(AT3G17609) TT4(AT5G13930) 2.3.1.74 27

EGL3(AT1G63650) TT4(AT5G13930) 2.3.1.74 27

MYB12(AT2G47460) TT4(AT5G13930) 2.3.1.74 27

GL3(AT5G41315) TT4(AT5G13930) 2.3.1.74 27

PIF3(AT5G13930) TT4(AT5G13930) 2.3.1.74 27

MYB75(AT1G56650) UGT78D2(AT5G17050) 2.4.1.- 12348

MYB32(AT4G34990) LDOX(AT4G22880) 1.14.11.23 67

**(d): TFs Phytohormone-biosynthetic enzyme gene Enzyme Crosstalk**

ATNFXL1(AT1G10170) EDS16(AT1G74710) 5.4.4.2 58

ATHB-1(AT3G01470) ATACO1(AT2G19590) 1.14.17.4 5*

EIN3(AT3G20770) EDS16(AT1G74710) 5.4.4.2 58

MYB30(AT3G28910) EDS16(AT1G74710) 5.4.4.2 58

MYB30(AT3G28910) EDS16(AT1G74710) 5.4.4.2 58

EIL1(AT2G27050) EDS16(AT1G74710) 5.4.4.2 58

**TFs Phytohormone-biosynthetic enzyme gene Enzyme Crosstalk**

OBP1(AT3G50410) GSTF8(AT2G47730) 2.5.1.18 578

AP1(AT1G69120) GSTF8(AT2G47730) 2.5.1.18 578

RAP2.3(AT3G16770) GSTF8(AT2G47730) 2.5.1.18 578

**(e): TFs Phytohormone-biosynthetic enzyme gene Enzyme Crosstalk**

MYB26(AT3G13890) IRX3(AT5G17420) 2.4.1.12 157

VND7(AT1G71930) IRX3(AT5G17420) 2.4.1.12 157

**TFs Phytohormone-biosynthetic enzyme gene Enzyme Crosstalk**

MYB34(AT5G60890) SUR1(AT2G20610) 2.6.1.5 127

MYB28(AT5G61420) SUR1(AT2G20610) 2.6.1.5 127

**TFs Phytohormone-biosynthetic enzyme gene Enzyme Crosstalk**

MYB29(AT5G07690) CYP79F2(AT1G16400) 1.14.13.- 37

EIL3(AT1G73730) CYP79F2(AT1G16400) 1.14.13.- 37

**TFs Phytohormone-biosynthetic enzyme gene Enzyme Crosstalk**

MYB58(AT1G16490) ATOMT1(AT5G54160) 2.1.1.- 26

MYB63(AT1G79180) ATOMT1(AT5G54160) 2.1.1.- 26

**Supplementary Information, section III: distinct TFs mediating multiple target enzymatic gene of various phytohormone biosynthesis**

**EIN3 (AT3G20770): ATEIN3, EIN3, ETHYLENE-INSENSITIVE3**

**Phytohormone-biosynthetic enzyme gene Enzyme Crosstalk**

ATACO2(AT1G62380) 1.14.17.4 5*

ATACO1(AT2G19590) 1.14.17.4 5*

EDS16(AT1G74710) 5.4.4.2 58

**PIL5 (AT2G20180): PHYTOCHROME INTERACTING FACTOR**

**Phytohormone-biosynthetic enzyme gene Enzyme Crosstalk**

NCED6(AT3G24220) 1.13.11.51 125

NCED9(AT1G78390) 1.14.99.- 157

NCED9(AT1G78390) 1.13.11.51 125

GA2OX2(AT1G30040) 1.14.11.- 567

GA3OX2(AT1G80340) 1.14.11.- 567

GA3OX1(AT1G15550) 1.14.11.- 567

GA2OX2(AT1G30040) 1.14.11.13 56

DWF4(AT3G50660) 1.14.13.- 37

CYP707A2(AT2G29090) 1.14.13.93 135

DWF4(AT3G50660) 1.14.13.93 135

AAO1(AT5G20960) 1.2.3.7 12

DFL1( AT5G54510) 6.3.-.- 127

**PIF3 (AT1G09530) , PHYTOCHROME INTERACTING FACTOR 3,**

**Phytohormone-biosynthetic enzyme gene Enzyme Crosstalk**

LDOX(AT4G22880) 1.14.11.- 567

LDOX(AT4G22880) 1.14.11.23 67

TT4(AT5G13930) 2.3.1.74 27

**LEC2 (AT1G28300)**

**Phytohormone-biosynthetic enzyme gene Enzyme Crosstalk**

GA3OX2(AT1G80340) 1.14.11.- 567

ACS4(AT2G22810 4.4.1.14 2457

**MYC2 AT1G32640 Other names: ATMYC2, JAI1, JASMONATE INSENSITIVE 1, JIN1, MYC2, RD22BP1, ZBF1**

**Phytohormone-biosynthetic enzyme gene Enzyme Crosstalk**

ADH1(AT1G77120) 1.1.1.1 58

LOX3(AT1G17420) 1.13.11.12 17

LOX2(AT3G45140) 1.13.11.12 17

**GL3 AT5G41315**

**Phytohormone-biosynthetic enzyme gene Enzyme Crosstalk**

LDOX(AT4G22880) 1.14.11.- 567

LDOX(AT4G22880) 1.14.11.23 67

FLS1(AT5G08640) 1.14.11.23 67

TT4(AT5G13930) 2.3.1.74 27

**ARF8(AT5G37020)**

**Phytohormone-biosynthetic enzyme gene Enzyme Crosstalk**

DFL1(AT5G54510) 6.3.-.- 127

GH3.17(AT1G28130) 6.3.-.- 127

**ARF7(AT5G20730)**

**Phytohormone-biosynthetic enzyme gene Enzyme Crosstalk**

BRU6(AT4G37390) 6.3.-.- 127

WES1(AT4G27260) 6.3.-.- 127

**HY5(AT5G11260)**

**Phytohormone-biosynthetic enzyme gene Enzyme Crosstalk**

LDOX(AT4G22880) 1.14.11.- 567

LDOX(AT4G22880) 1.14.11.23 67

TT4(AT5G13930) 2.3.1.74 27

**EGL3(AT1G63650)**

**Phytohormone-biosynthetic enzyme gene Enzyme Crosstalk**

LDOX(AT4G22880) 1.14.11.- 567

FLS1(AT5G08640) 1.14.11.23 67

LDOX(AT4G22880) 1.14.11.23 67

TT4(AT5G13930) 2.3.1.74 27

**STY1(AT3G51060)**

**Phytohormone-biosynthetic enzyme gene Enzyme Crosstalk**

WES1(AT4G27260) 6.3.-.- 127

GH3.4(AT1G59500) 6.3.-.- 127

GH3.3(AT2G23170) 6.3.-.- 127

DFL1(AT5G54510) 6.3.-.- 127

BRU6(AT4G37390) 6.3.-.- 127
